# Regulatory T-lymphocytes mediate amyotrophic lateral sclerosis progression and survival

**DOI:** 10.1002/emmm.201201544

**Published:** 2012-11-09

**Authors:** Jenny S Henkel, David R Beers, Shixiang Wen, Andreana L Rivera, Karen M Toennis, Joan E Appel, Weihua Zhao, Dan H Moore, Suzanne Z Powell, Stanley H Appel

**Affiliations:** 1 Department of Neurology, Methodist Neurological Institute, The Methodist Hospital Research InstituteHouston, TX, USA; 2 Department of Pathology, The Methodist HospitalHouston, TX, USA; 3 Forbes-Norris ALS Center, California Pacific Medical CenterSan Francisco, CA, USA

**Keywords:** ALS, FoxP3, Gata3, survival, Tregs

## Abstract

In amyotrophic lateral sclerosis (ALS) mice, regulatory T-lymphocytes (Tregs) are neuroprotective, slowing disease progression. To address whether Tregs and FoxP3, a transcription factor required for Treg function, similarly influence progression rates of ALS patients, T-lymphocytes from patients were assessed by flow cytometry. Both numbers of Tregs and their FoxP3 protein expressions were reduced in rapidly progressing ALS patients and inversely correlated with progression rates. The mRNA levels of FoxP3, TGF-β, IL4 and Gata3, a Th2 transcription factor, were reduced in rapidly progressing patients and inversely correlated with progression rates. Both FoxP3 and Gata3 were accurate indicators of progression rates. No differences in IL10, Tbx21, a Th1 transcription factor or IFN-γ expression were found between slow and rapidly progressing patients. A 3.5-year prospective study with a second larger cohort revealed that early reduced FoxP3 levels were indicative of progression rates at collection and predictive of future rapid progression and attenuated survival. Collectively, these data suggest that Tregs and Th2 lymphocytes influence disease progression rates. Importantly, early reduced FoxP3 levels could be used to identify rapidly progressing patients.

## INTRODUCTION

Although neuroinflammation is a pathological feature in amyotrophic lateral sclerosis (ALS) patients, its role in the pathogenic process is still under investigation. Multiple studies have addressed T-lymphocyte infiltration in the central nervous system (CNS) of ALS patients; lymphocytes were found in the majority of patient spinal cords and along the vessels in the pre-central gyrus extending into the areas of neuronal injury (Graves et al, 2004; Kawamata et al, 1992; Lampson et al, 1990; Troost et al, 1990, 1989). Our investigations found perivascular and intraparenchymal CD4^+^ T-lymphocytes in the proximity of degenerating corticospinal tracts and ventral horns in two-thirds of ALS patients (Engelhardt et al, 1993). In the blood of ALS patients, alterations in T-lymphocyte populations have also been described as compared with controls (Lincecum et al, 2010; Mantovani et al, 2009; Seksenyan et al, 2010; Shi et al, 2007; Zhang et al, 2005); however, these studies do not address whether T-lymphocytes directly or indirectly influence disease progression.

The innate and adaptive immune systems play pivotal and interdependent roles regulating the rate of disease progression in ALS mice overexpressing mutant superoxide dismutase 1 (mSOD1; Appel et al, 2010; Banerjee et al, 2008; Beers et al, 2006, 2008, 2011a; Boillée et al, 2006; Chiu et al, 2008; Henkel et al, 2009). We and others demonstrated that CD4^+^ T-lymphocytes slowed disease progression with a 50% increase in disease duration, modified the microglial phenotypes, and extended survival (Beers et al, 2008, 2011a; Chiu et al, 2008). Banerjee et al. also concluded that *ex vivo* activated CD4^+^ T-lymphocytes improved neurological function and survival of ALS mice (Banerjee et al, 2008). One population of T-lymphocytes upregulated during the stable phase in ALS mice (Beers et al, 2011a) and which could be involved in slowing disease progression of ALS patients are CD4^+^CD25^High^FoxP3^+^ regulatory T-lymphocytes (Tregs). Tregs suppress both innate and adaptive immune reactions detrimental to the host, down-regulate pro-inflammatory cytokine production, and can suppress the activation/expansion of CD4^+^CD25^−^ effector T-lymphocytes (Teffs). Recently, Tregs have been shown to directly steer the differentiation of macrophages and microglia toward an alternative M2 activation state (Li et al, 2010; Liu et al, 2011; Mahnke et al, 2007; Reynolds et al, 2009a, b), and in turn, M2 cells induce Tregs which suppress Teffs (Cools et al, 2008; Mahnke et al, 2007; Savage et al, 2008). Since Tregs are critically involved in suppressing inflammation induced by neurotoxic T-lymphocytes and microglia/macrophages, and since they play a prominent role in slowing the rate of progression in ALS mice (Banerjee et al, 2008; Beers et al, 2008, 2011a; Chiu et al, 2008), their potential involvement in modifying disease progression was investigated in patients with ALS.

## RESULTS

### Treg numbers and FoxP3 expression decrease as ALS progression rates increase

**Figure 1:**
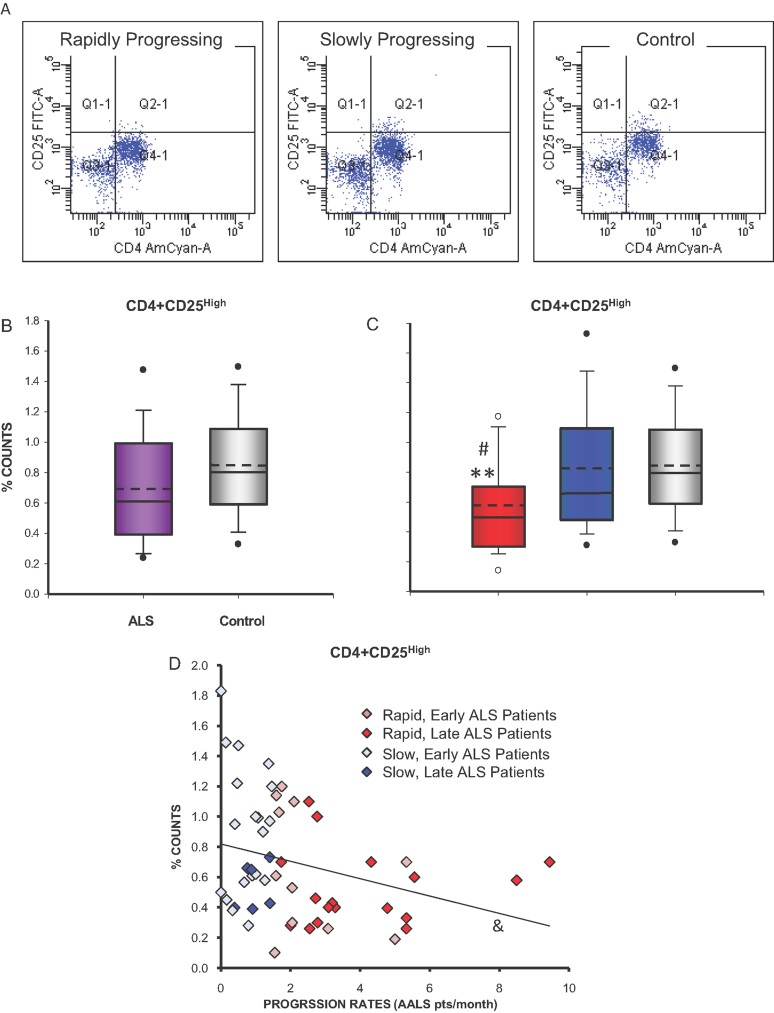
CD4^+^CD25^High^ regulatory T-lymphocytes (Tregs) are reduced in rapidly progressing ALS patients Shown are flow cytometric analyses of leukocytes from 54 ALS patients through all stages of disease and 33 control volunteers. Representative flow diagrams showing CD4^+^CD25^High^ Tregs from a rapidly progressing ALS patient, a slowly progressing ALS patient, and a control volunteer.Box and whisker plots indicating that percent of CD4^+^CD25^High^ Tregs in total leukocytes from ALS patients (mean = 0.692%, median = 0.610%) were not different when compared with control volunteers using the *t*-test (mean = 0.845%, median = 0.800%).When the ALS patients were separated based on the rate of disease progression into rapidly (AALS points per month ≥1.5; 26 patients) *versus* slowly (AALS points per month <1.5; 28 patients) progressing ALS patients, the percent of CD4^+^CD25^High^ Tregs were reduced in rapidly progressing patients (mean = 0.573%, median = 0.495%) compared with slowly progressing patients (mean = 0.825%, median = 0.660%) and reduced compared with control volunteers (mean = 0.845%, median = 0.800%); slowly progressing patients were not different than controls. ^#^*p* = 0.018 *versus* slowly progressing ALS patients; **p = 0.003 *versus* controls.Scatter plot with regression line demonstrating that the percent of CD4^+^CD25^High^ T cells were inversely correlated with rate of ALS progression (*R* = 0.301; linear regression). Slowly progressing ALS patients = AALS points/month <1.5; rapidly progressing ALS patients = AALS points/month >1.5, at the time of collection. ALS patients early in disease = AALS score < 100; ALS patients late in disease = AALS score ≥100, at the time of collection. ^&^*p* = 0.028.

CD4^+^CD25^High^ Tregs were evaluated to determine whether they are associated with disease progression in ALS patients, as in mSOD1 mice. Single blood samples from 54 ALS patients from all stages of disease and control volunteers were examined by flow cytometry (Fig 1A). The percentages of CD3^+^, CD4^+^ or CD8^+^ lymphocytes (data not shown), or CD4^+^CD25^High^ Tregs (Fig 1B) of total leukocytes from ALS patients and controls were not different. However, differences were noted when the patients were separated and grouped based on their rate of disease progression at the time of blood collection [rate was determined as the change in AALS score, change in time (months), comparing the initial clinical evaluation with the evaluation performed at the time of blood collection]. Patients were grouped into slowly (<1.5 AALS points/month, 28 patients) *versus* rapidly (≥1.5 AALS points/month, 26 patients) progressing patients using the Appel ALS (AALS) score (Fig 1C; Haverkamp et al, 1995), as this rate separated the patients surviving only a year or two after diagnosis from those that survive up to 6 years or more after diagnosis and also divided these patients approximately in half. Leukocytes from blood of rapidly progressing patients consisted of 31% fewer CD4^+^CD25^High^ Tregs compared with slowly progressing patients (*p* = 0.018) and 32% fewer CD4^+^CD25^High^ Tregs compared with controls (*p* = 0.003). CD4^+^CD25^High^ Tregs from slowly progressing patients were not different than controls (*p* = 0.846). No correlation with age (*p* = 0.689) nor difference between limb *versus* bulbar onsets (*p* = 0.684) were found. The percent of CD4^+^CD25^High^ Tregs in ALS patients were inversely correlated with rate of disease progression; the greater the number of Tregs, the slower the disease progression rate (*p* = 0.028; Fig 1D). These results indicate an association between numbers of CD4^+^CD25^High^ Tregs in blood with the rate at which disease progresses in ALS patients.

**Figure 2:**
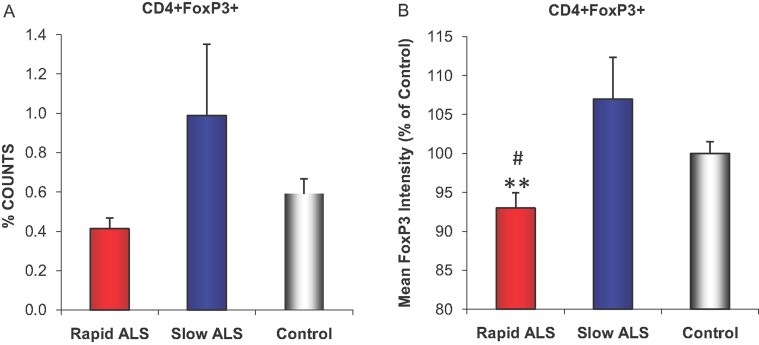
FoxP3 intensity in CD4^+^FoxP3^+^ Tregs is reduced in rapidly progressing ALS patients There was a trend toward reduced numbers of CD4^+^FoxP3^+^ Tregs in the blood of rapidly progressing patients compared with both slowly progressing patients and control volunteers.FoxP3 intensity in CD4^+^FoxP3^+^ Tregs is reduced in rapidly progressing patients compared with slowly progressing patients and compared with control volunteers; slowly progressing patients were not different than controls. ^#^*p* = 0.049 *versus* slowly progressing ALS patients; **p = 0.015 *versus* controls.

The Treg transcription factor, FoxP3, is currently the most reliable marker for identifying Tregs. Therefore, CD4^+^FoxP3^+^ Tregs from the same 54 ALS patients were evaluated to determine whether CD4^+^FoxP3^+^ Tregs were also associated with disease progression. While not significant, there were trends toward reduced CD4^+^FoxP3^+^ Tregs in rapidly progressing patients compared with controls (*p* = 0.082) and slowly progressing patients (*p* = 0.196; Fig 2A). CD4^+^FoxP3^+^ Tregs from slowly progressing patients were not different than controls (*p* = 0.371). High FoxP3 expression is required for the suppressive function of Tregs (Sakaguchi et al, 2010); therefore, we also examined FoxP3 fluorescent intensity by flow cytometry, as an assessment of FoxP3 protein expression, in CD4^+^FoxP3^+^ Tregs. The FoxP3 fluorescent intensities in CD4^+^FoxP3^+^ Tregs were reduced in rapidly progressing patients compared with controls (*p* = 0.015; Fig 2B) and slowly progressing patients (*p* = 0.049) and inversely correlated with progression rates (*p* = 0.047). The FoxP3 fluorescent intensities in Tregs from slowly progressing patients were not different than controls (*p* = 0.296). Thus, as with CD4^+^CD25^High^ Tregs numbers, there was a reduction in FoxP3 fluorescent intensity in CD4^+^FoxP3^+^ Tregs from rapidly progressing ALS patients.

**Figure 3:**
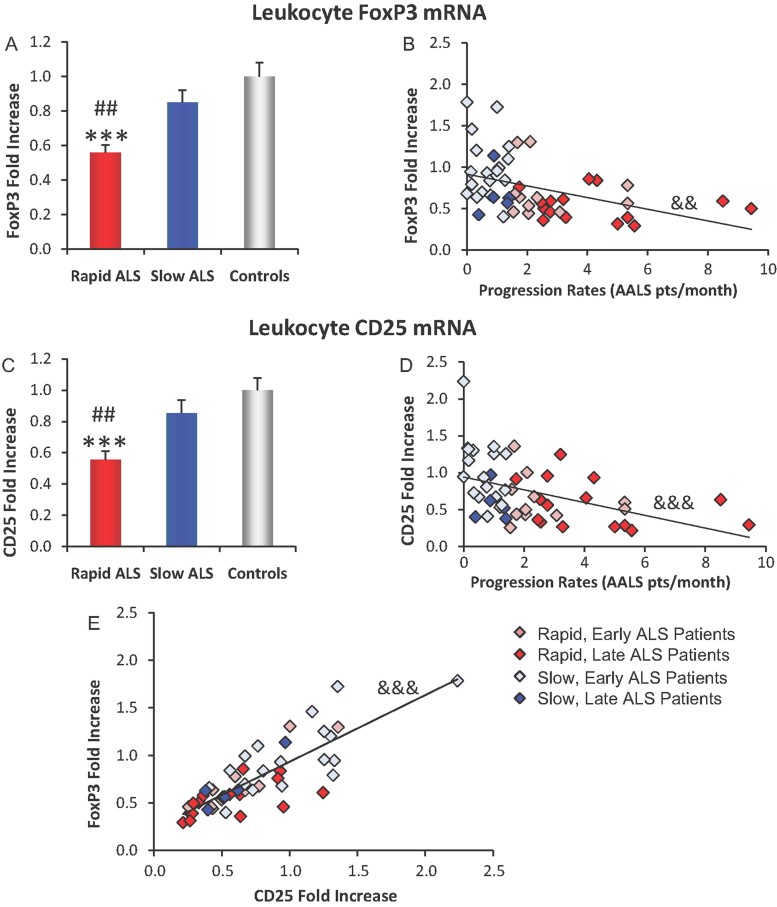
Leukocyte FoxP3 and CD25 mRNA expression levels are reduced in rapidly progressing ALS patients qRT-PCR was utilized to evaluate mRNA expression levels of FoxP3 and CD25 in leukocytes obtained from 54 ALS patients through all stages of disease and 33 control volunteers. **A,B.** FoxP3 mRNA expression levels were down-regulated in rapidly progressing ALS patients (*t*-test) and negatively correlated with disease progression rates (*R* = 0.419; linear regression).**C,D.** CD25 mRNA expression levels were reduced in rapidly progressing ALS patients (*t*-test) and inversely correlated with rate of disease progression (*R* = 0.444; linear regression).**E.** FoxP3 and CD25 mRNA expression levels of ALS patients directly correlated with each other (*R* = 0.815; linear regression). Note that slowly progressing patients early in their disease expressed the highest FoxP3 and CD25 mRNA levels, whereas rapidly progressing patients late in their course of disease expressed the lowest levels of FoxP3 and CD25 mRNAs. ^##^*p* ≤ 0.005 *versus* slowly progressing ALS patients. ***p ≤ 0.001 *versus* controls. ^&&^*p* ≤ 0.005, ^&&&^*p* ≤ 0.001.

As an independent assessment, leukocyte FoxP3 and CD25 mRNA expression levels were also examined by quantitative (q)RT-PCR. FoxP3 mRNA levels from rapidly progressing patients were reduced 34% compared with slowly progressing patients (*p* = 0.001) and 44% compared with controls (*p* = 0.00002; Fig 3A). CD25 mRNA levels in leukocytes from rapidly progressing ALS patients were reduced 35% compared with slowly progressing patients (*p* = 0.006) and 45% compared with controls (*p* = 0.00003; Fig 3C). FoxP3 and CD25 mRNA levels in slowly progressing patients were not different than controls (*p* = 0.164, *p* = 0.211, respectively). Both the FoxP3 and CD25 expression levels in patients were inversely correlated with rates of progression (*p* = 0.003, *p* = 0.001, respectively; Fig 3B and D). CD25 mRNA expression levels directly correlated with FoxP3 mRNA levels (*p* < 0.001; Fig 3E); note that rapidly progressing patients late in their course of disease expressed the lowest levels of FoxP3 and CD25 mRNAs, whereas slowly progressing patients early in their disease expressed the highest FoxP3 and CD25 mRNA levels.

### Leukocytes from rapidly progressing patients expressed low mRNA levels of the Th2 transcription factor Gata3 and the anti-inflammatory cytokines IL4 and TGF-β, yet no difference in IFN-γ or the Th1 transcription factor Tbx21

**Figure 4:**
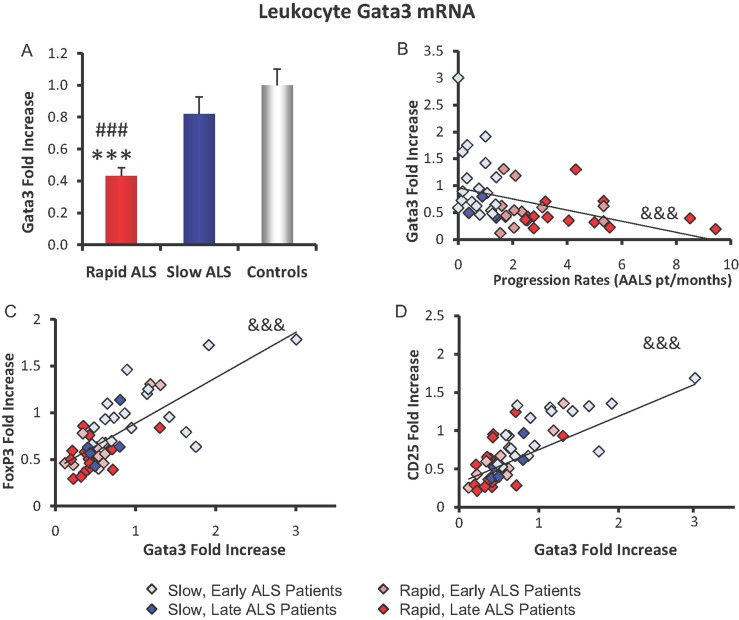
Leukocyte Gata3 mRNA expression levels are reduced in rapidly progressing ALS patients qRT-PCR was utilized to evaluate expression levels of Gata3 mRNA isolated from leukocytes of ALS patients and control volunteers. **A,B.** Gata3 mRNA expression was decreased in rapidly progressing ALS patients (*t*-test) and inversely correlated with rate of disease progression (*R* = 0.405; linear regression).**C.** Gata3 and FoxP3 levels correlated (*R* = 0.737; linear regression).**D.** Gata3 and CD25 mRNA levels correlated (*R* = 0.810; linear regression). Note that slowly progressing patients early in their disease expressed the highest Gata3 mRNA levels, whereas rapidly progressing patients late in their course of disease expressed the lowest levels of Gata3 levels. ^###^*p* = 0.003 *versus* slowly progressing ALS patients; ***p = 0.00001 *versus* controls; ^&&&^*p* ≤ 0.004.

As Th2 lymphocytes are also neuroprotective in the ALS mouse (Beers et al, 2011b), we addressed whether Gata3, the master transcription factor for Th2 lymphocytes (Zhu & Paul, 2010) might also be affected. Gata3 levels in rapidly progressing patients were reduced 47% compared with slowly progressing patients (*p* = 0.003) and 57% compared with control volunteers (*p* = 0.00001; Fig 4A). Gata3 levels in ALS patients' leukocytes were inversely correlated with disease progression rates (*p* = 0.004; Fig 4B). Gata3 levels also directly correlated with FoxP3 and CD25 levels (both *p* < 0.001; Fig 4C and D).

**Figure 5:**
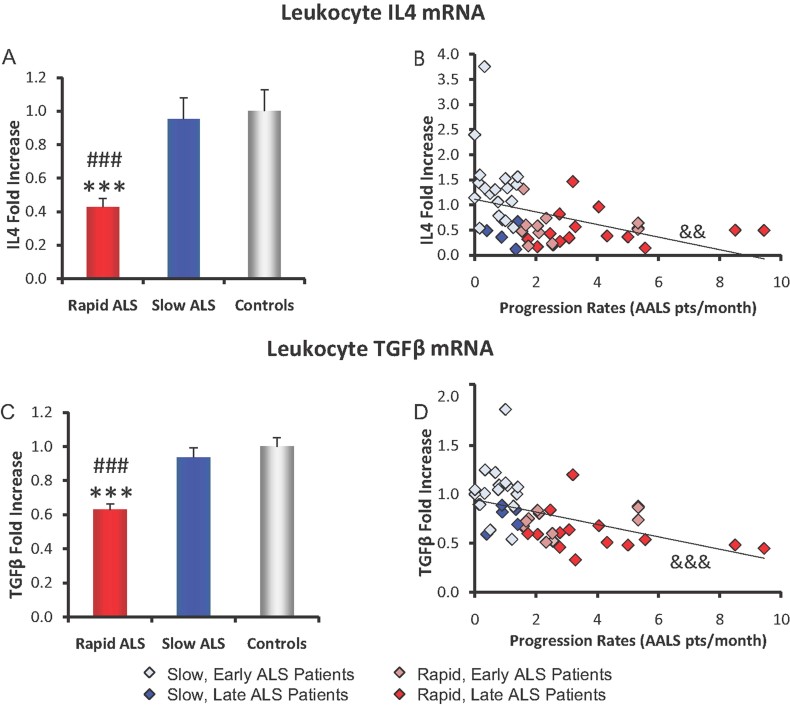
Leukocyte IL4 and TGF-β mRNA expression levels are reduced in rapidly progressing ALS patients qRT-PCR was utilized to evaluate mRNA expression levels of IL4 and TGF-β in leukocytes obtained from 54 ALS patients through all stages of disease and 33 control volunteers. **A,B.** IL4 mRNA expression was decreased in rapidly progressing ALS patients (*t*-test) and inversely correlated with rate of disease progression (*R* = 0.388; linear regression).**C,D.** TGF-β mRNA expression was reduced in rapidly progressing ALS patients (*t*-test) and inversely correlated with rate of disease progression (*R* = 0.475; linear regression). Note that slowly progressing patients early in their disease expressed the highest IL4 and TGF-β mRNA levels, whereas rapidly progressing patients late in their course of disease expressed the lowest levels of IL4 and TGF-β levels. ^###^*p* ≤ 0.0007 *versus* slowly progressing ALS patients; ***p ≤ 0.0002 *versus* controls; ^&&^*p* ≤ 0.006, ^&&&^*p* ≤ 0.001.

As Tregs and Th2 lymphocytes can both express anti-inflammatory cytokines, we evaluated TGF-β, IL4 and IL10 mRNA levels and whether they might also correlate with progression rates. IL4 expression levels in rapidly progressing patients were reduced 55% compared with slowly progressing patients (*p* = 0.0007) and 57% compared with controls (*p* = 0.0002; Fig 5A). TGF-β expression levels in rapidly progressing patients were reduced 33% compared with slowly progressing patients (*p* = 0.00003) and 37% compared with controls (*p* = 0.0000004; Fig 5C). IL4 and TGF-β levels were not different between slowly progressing patients and controls (*p* = 0.792, *p* = 0.408, respectively). IL4 and TGF-β mRNA levels inversely correlated with progression rates (*p* = 0.006, *p* < 0.001, respectively; Fig 5B and D). Two ALS patients had an AALS rate of zero points/month: one of the two patients had the highest CD25 and FoxP3 expression levels and the other patient had one of the highest IL4 levels. There were trends toward reduced IL10 expression in rapidly progressing patients compared with slowly progressing patients (*p* = 0.161) or compared with controls (*p* = 0.073; Supporting Information Fig 1A), but no correlation with progression rate was found (*p* = 0.285; Supporting Information Fig 1B).

IL4 mRNA levels directly correlated with FoxP3, CD25 and Gata3 levels (*p* = 0.003, *p* < 0.001, *p* = 0.027, respectively; Supporting Information Fig 2B, D and F), and TGF-β mRNA levels directly correlated with FoxP3, CD25 and Gata3 levels (all three *p* < 0.001; Supporting Information Fig 2A, C and E). There were only trends toward correlations between IL10 and FoxP3 or Gata3 mRNA levels (*p* = 0.115, *p* = 0.185, respectively; Supporting Information Fig 1C and E); IL10 mRNA levels did not correlate with CD25 (*p* = 0.338; Supporting Information Fig 1D). To ensure that not every factor was reduced in rapidly progressing patients, we also examined mRNA levels for the master Th1 lymphocyte transcription factor, Tbx21 (Tbet) and for their prototypic pro-inflammatory cytokine, IFN-γ. No differences in Tbx21 or IFN-γ levels in ALS patients' leukocytes were observed compared with controls (Supporting Information Fig 3A and C) and there were no correlations with disease progression rates (Supporting Information Fig 3B and D).

### Levels of anti- and pro-inflammatory factors in ALS spinal cord

**Figure 6:**
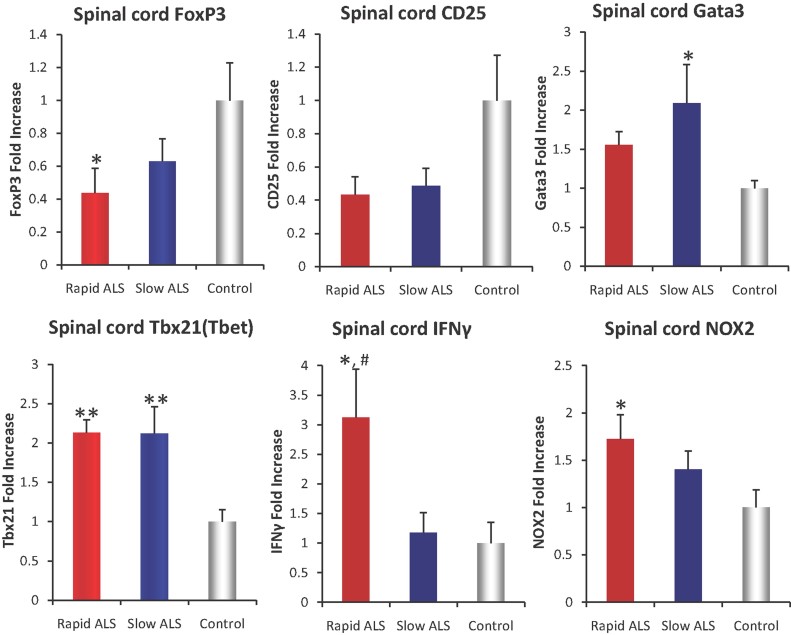
FoxP3, CD25, Gata3, TGF-β, IL4, IL10, Tbx21 (Tbet), IFN-γ and NOX2 mRNA expression levels in spinal cord autopsy tissue from ALS patients qRT-PCR was utilized to evaluate mRNA expression levels of CD25, FoxP3, Gata3 (Th2 transcription factor), Tbx21 (Th1 transcription factor), IFN-γ and NOX2 in spinal cord autopsy tissue obtained from 34 ALS patients and 14 disease control. Spinal cord FoxP3 mRNA expression levels were reduced in ALS patients who had progressed rapidly, CD25 mRNA expression levels were not significantly reduced, and Gata3 mRNA expression levels of ALS patients were increased in ALS patients who had progressed slowly. Spinal cord Tbx21 mRNA expression levels were increased in ALS patients who had progressed slowly or rapidly, IFN-γ levels were upregulated in patients who had progressed rapidly, and NOX2 levels were increased in patients who had progressed rapidly (*t*-test). Therefore, while Tbx21 levels in spinal cord were upregulated in patients who had progressed either rapidly or slowly *versus* controls, IFN-γ and NOX2 levels were upregulated only in patients who had progressed rapidly, suggesting an active suppression as observed in the mSOD1 mouse. *p ≤ 0.05 *versus* controls, **p ≤ 0.01 *versus* controls, ^#^*p* ≤ 0.05 *versus* slowly progressing.

To address whether the low FoxP3 and Gata3 expressions in the peripheral leukocytes of rapidly progressing patients might be due to enhanced infiltration of Treg and Th2 lymphocytes from the periphery into the spinal cord tissue and address whether the infiltrating T-lymphocytes could potentially influence disease progression rates, mRNA levels were examined in postmortem spinal cord from 15 patients who had progressed rapidly, 19 patients who had progressed slowly and 14 controls and compared with the patients' prior progression rates (Fig 6). FoxP3 mRNA levels were reduced 56.2% in patients who had progressed rapidly compared with disease controls (*p* = 0.042), and Gata3 levels were enhanced 108.7% in patients who had progressed slowly (*p* = 0.045) compared with controls. Therefore, while these analyses were performed on tissues from patients after death, the reduced FoxP3 and Gata3 expressions in peripheral leukocytes of patients who had progressed rapidly do not appear to be due to an enhanced influx of Tregs and Th2 lymphocytes into the CNS. Additionally, Tbx21 mRNA levels in spinal cord were similarly increased in patients who had progressed rapidly (113.2%) or slowly (112.1%) *versus* controls (*p* = 0.0084, *p* = 0.0079, respectively). IFN-γ levels were upregulated 212.0% in patients who had progressed rapidly compared with controls (*p* = 0.024) and compared with slowly progressing patients (*p* = 0.036). There was no difference in IFN-γ levels between slowly progressing patients and controls (*p* = 0.724). NOX2 levels were upregulated 72.5% in patients who had progressed rapidly compared with controls (*p* = 0.028). This similar increase in Tbx21 expression in patients who had progressed slowly and rapidly compared with controls, yet no corresponding upregulation of IFN-γ mRNA in the patients who had progressed slowly, suggest an active suppression of IFN-γ expression in Th1 cells in slowly progressing patients as was observed in the mSOD1 mouse during the slow phase (Beers et al, 2011a).

### FoxP3 and Gata3 expression levels: potential indicators of disease progression rates

**Figure 7:**
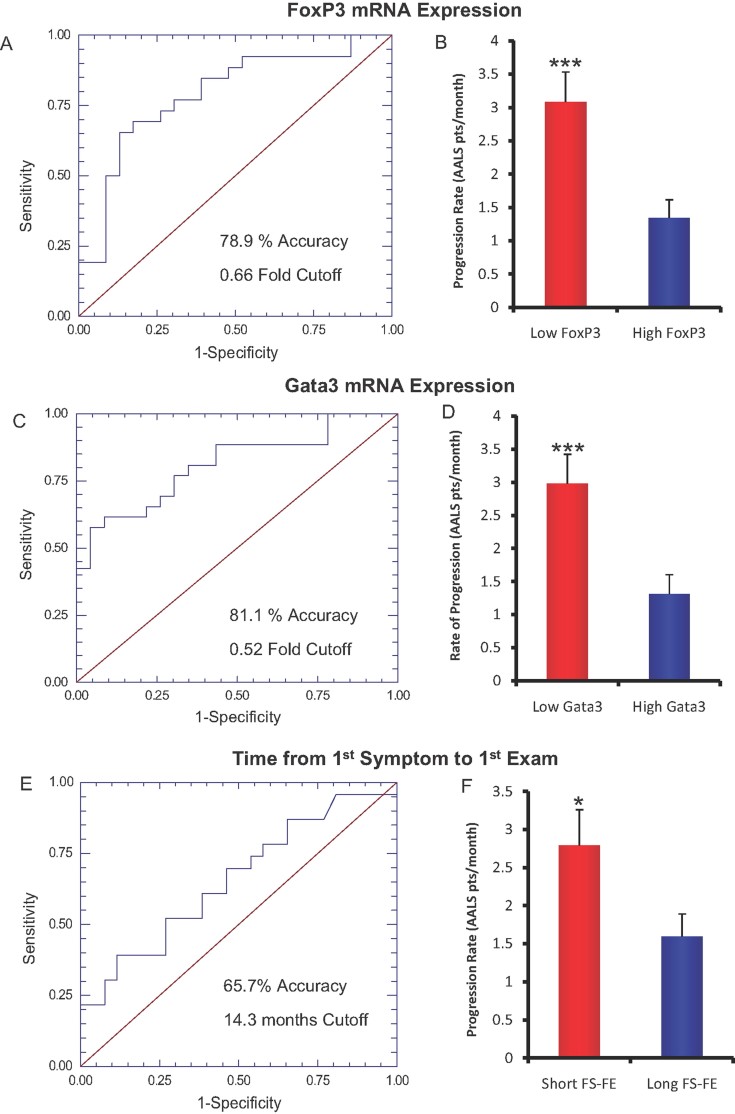
FoxP3 and Gata3 expression levels are potential predictors of ALS progression rates Shown are the receiver operating characteristic (ROC) analyses of FoxP3 and Gata3 mRNA expression in 54 patients compared to their progression rates depicted in Fig and 4: the ROC analyses ‘training’ data sets. FoxP3 expression had a 78.9% accuracy (95% CI: 0.658–0.921), 73.9% sensitivity and 73.1% specificity, using a fold-increase over 0.66 as positive and a rate of progression of less than 1.5 points per month as slow.ALS patients with low FoxP3 mRNA expression levels (based on the 0.66-fold cutoff determined by the ROC analysis) progressed more rapidly than patients with high FoxP3 levels.Gata3 expression had a 81.1% accuracy (95% CI: 0.651–0.902), 76.9% sensitivity and 69.9% specificity, using a fold-increase over 0.52 as positive and a rate of progression of less than 1.5 points per month as slow.ALS patients with low Gata3 mRNA expression levels (based on the 0.52-fold cutoff determined by the ROC analysis) progressed more rapidly than patients with high Gata3 levels.Also shown for comparison is the ROC analysis of the time from first symptom to first exam in the same 54 patients compared to their progression rates. Time from first symptom to first exam had a 65.7% accuracy (95% CI: 0.473–0.786), 60.9% sensitivity and 61.5% specificity, using a fold-increase over 14.3 months as positive and a rate of progression of less than 1.5 points per month as slow.ALS patients with short times from first symptom to first exam (based on the 14.3 months cutoff determined by the ROC analysis) progressed more rapidly than patients with longer times from first symptom to first exam. *p = 0.04; ***p = 0.003.

The correlations between leukocyte FoxP3 or Gata3 mRNA expression levels and disease progression rates prompted our evaluation of the mRNA levels as potential indicators of the patient's current clinically assessed disease progression rate and as potential predictors of future progression rates. Receiver operating characteristic (ROC) analyses (Fig 7A and C) were used to evaluate the accuracy of mRNA levels from these 54 patients for reflecting slow *versus* rapid disease progression rates at the time of leukocyte collection (using both the mRNA levels and the rate of progression as previously shown and described in Fig and 4 as the ROC analyses ‘training’ data set; *Slow*: <1.5 AALS points per month). FoxP3 mRNA levels were an accurate indicator of disease progression rates. Using a ROC cutoff over 0.66-fold of control as positive, FoxP3 expression had a 78.9% accuracy, 73.9% sensitivity and 73.1% specificity. Grouping the patients using the 0.66-fold cutoff, patients expressing low FoxP3 progressed 2.29-fold more rapidly than patients with high FoxP3 levels (*p* = 0.003; Fig 7B). Gata3 mRNA levels were also an accurate indicator of disease progression rates. Using a ROC cutoff over 0.52-fold of control as positive, Gata3 expression had an 81.1% accuracy, 76.9% sensitivity and 69.6% specificity. Grouping the patients using the 0.52-fold cutoff, patients expressing low Gata3 progressed 2.27-fold more rapidly than patients with high GataP3 (*p* = 0.003; Fig 7D).

For comparison, the accuracy of a currently used prognostic indicator of future ALS progression rate, the time from first symptom to first examination, was evaluated using the same 54 patients. Patients with a shorter time from first symptom to first examination tend to progress more rapidly than patients with a longer time from first symptom to first exam (Czaplinski et al, 2006; Kollewe et al, 2008). A ROC analysis was used to evaluate the accuracy of the time from first symptom to first exam for reflecting disease progression rates at the time of collection (Fig 7E). Using a ROC cutoff over 14.3 months as positive, time from first symptom to first exam had a 65.7% accuracy, a 60.9% sensitivity and a 61.5% specificity. Grouping the patients using the 14.3 months cutoff, patients with a shorter time from first symptom to first exam progressed 75.3% more rapidly than patients with longer time from first symptom to first exam (*p* = 0.04; Fig 7F). Therefore, although the time from first symptom to first exam was reflecting disease progression rates, FoxP3 and Gata3 mRNA expression levels were more accurate indicators of progression rates than time from first symptom to first exam.

### Analyses of a second group of ALS patients verified the inverse correlation of FoxP3 expression with disease progression rates; patients with low FoxP3 levels progressed more rapidly and low FoxP3 expression levels were predictive of future rapid progression rates

**Figure 8:**
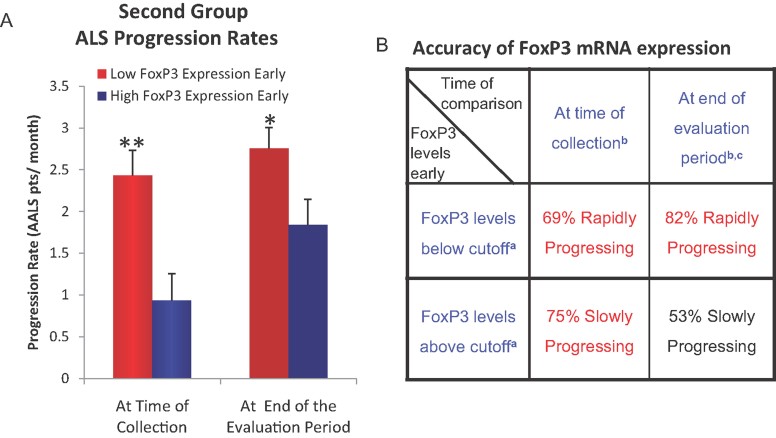
FoxP3 mRNA expression levels in a second group of ALS patients were reflective of progression rates at the time of collection, and low FoxP3 levels predicted future rapid progression rates Leukocyte mRNAs from 102 patients were collected over a 3-year period during the early stages of disease and evaluated for FoxP3 mRNA expression levels. These levels were compared with progression rates both at the time of collection and at the end of the evaluation period (3.5 years). With this new ‘test’ set of 102 patients, ALS patients with low FoxP3 mRNA expression levels (based on cutoffs determined by the ROC analysis of the original 54 patients) early in disease progressed more rapidly than patients with high FoxP3 levels, both at the time of collection and at the end of the evaluation period. *p < 0.05 *versus* high FoxP3 levels, **p < 0.01 *versus* high FoxP3 levels.FoxP3 levels of this new ‘test’ set of patients were reflective of progression rates at the time of collection and low FoxP3 levels were predictive of future rapid progression rates. At the time of collection, low FoxP3 levels correctly predicted rapid progression rates 69% of the time, and high FoxP3 levels correctly predicted slow progression rates 75% of the time. At the end of the analysis period, low FoxP3 levels correctly predicted rapid progression rates 82% of the time, and high FoxP3 levels correctly predicted slow progression rates 53% of the time. ^a^Cutoff levels were defined by the prior ROC curve analyses (Fig 7; FoxP3 = 0.66). ^b^Levels were compared with progression rates both at the time of collection and at the end of the evaluation period. ^c^Evaluation period = 3.5 years.

The above analyses were performed on leukocytes from the 54 ALS patients obtained at all stages of disease and compared with their respective progression rates at the time of collection. For an analysis of FoxP3 levels or Gata3 levels to be of benefit in the clinic, they would need to predict future progression rates. Therefore, to evaluate whether early FoxP3 or Gata3 levels might be predictive of future disease progression rates, leukocyte RNAs from a single time-point were collected from a second group of 102 ALS patients during their early stages of disease; their AALS scores were re-evaluated every 3 months over a 3.5-year evaluation period to determine the time to 100 AALS points and survival (‘test’ data set). As with the prior analyses, patients expressing low FoxP3 levels early in disease were progressing more rapidly than patients expressing high FoxP3 levels at the time of collection (*p* = 0.00095; Fig 8A), and early FoxP3 levels inversely correlated with disease progression rates at the time of collection (*p* = 0.012). On an individual basis, early FoxP3 levels accurately reflected progression rates at the time of collection and verified our observations with the first patient group: 75% of all patients with FoxP3 levels above the cutoff were slowly progressing at the time of collection; 69% of all patients with FoxP3 levels below the cutoff were rapidly progressing at the time of collection (Fig 8B). These results not only verified our previous results that FoxP3 levels accurately reflect progression rates for the short-term but also indicated that FoxP3 levels can be used as an additional measure to monitor disease progression.

Similar analyses were performed evaluating Gata3 expression. While Gata3 mRNA levels were an accurate indicator of progression when patients were from all stages of disease, they were not accurate when samples were obtained only from patients at early stages of disease. Most patients (83%) expressed high Gata3 levels early in disease and there was no difference in Gata3 levels in the three groups. Hence, patients expressing low Gata3 mRNA levels early in disease were not progressing more rapidly than patients expressing high Gata3 (*p* = 0.259), and early Gata3 levels were not correlated with disease progression rates at the time of collection (*p* = 0.172). Therefore, the high Gata3 mRNA levels expressed by most patients early in disease limit the usefulness of early Gata3 levels as a predictive indicator.

To evaluate whether early FoxP3 levels could be used to predict future progression rates, the mRNA levels in leukocytes from the 102 patients obtained early in disease were compared with their rate of progression at the end of the evaluation period [rate was determined as the change in AALS score, change in time (months), comparing the initial evaluation with the evaluation at the end of the 3.5-year follow-up period]. As observed at the time of collection, patients expressing low FoxP3 levels early in disease were progressing more rapidly at the end of the analysis period than patients expressing high FoxP3 levels (*p* = 0.025; Fig 8A). Additionally, early low FoxP3 levels accurately predicted progression rates at the end of the analysis period: 82% of patients with FoxP3 levels below the cutoff were rapidly progressing at the end of the evaluation period (Fig 8B). The increased predictability of FoxP3 levels below the cutoff, comparing the time of collection *versus* the end of analysis period, was due to 40% of the slowly progressing patients, with low FoxP3 levels at the time of collection, progressing rapidly shortly thereafter. While high FoxP3 levels were reflective of progression rates at the time of blood collection, they were not predictive of future progression rates. Of the patients with FoxP3 levels above the cutoffs, 53% were slowly progressing at the end of the evaluation period (Fig 8B). The reason for the lack of predictability of FoxP3 levels above the cutoff was that 43% of the patients expressing high FoxP3 levels who were slowly progressing at the time of collection began rapidly progressing during the 3.5-year evaluation period. Therefore, low FoxP3 levels are highly predictive of future rapidly progressing disease, while high levels do not predict future slowly progressing disease, as the time-span the patient will remain slowly progressing is variable.

### Low FoxP3 expression levels predict an attenuated survival

**Figure 9:**
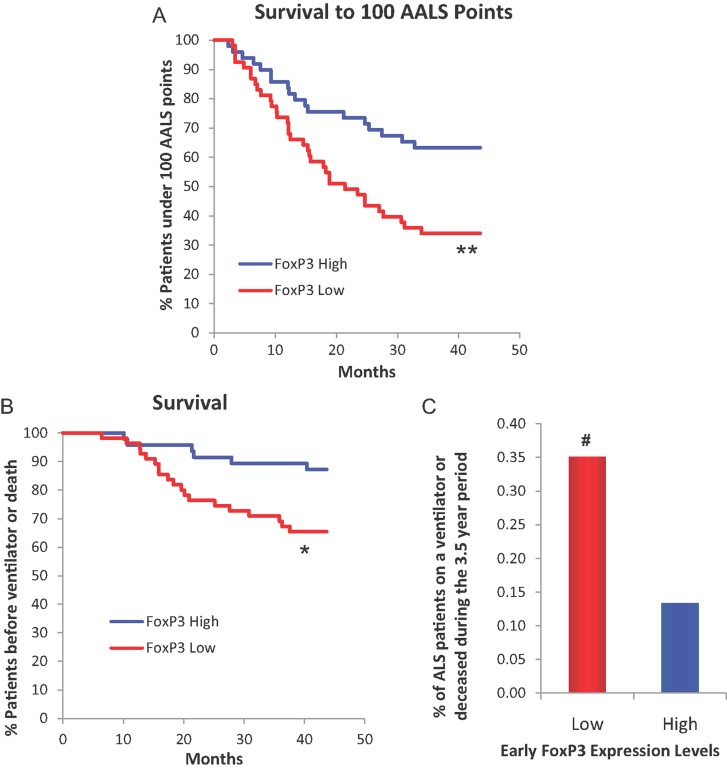
Low FoxP3 expression levels are predictive of reduced survival Leukocytes from 102 patients were collected over a 3-year period during the early stages of disease and evaluated for FoxP3 mRNA expression levels. AALS score was determined every 3 months for 3.5 years. **A.** Sixty-six percent of patients with FoxP3 expression levels below the cutoff were above 100 AALS points at the end of the 3.5 years, while only 36.7% of the patients with FoxP3 expression levels above the cutoff were above 100 AALS points.**B,C.** Thirty-five percent of patients with FoxP3 expression levels below the cutoff were placed on a ventilator or were deceased during the 3.5 years, while only 13% of the patients with FoxP3 expression levels above the cutoff were on a ventilator or deceased. Optimum cutoff level was defined by the ROC curve analysis in Fig 7 (FoxP3 = 0.66). *p = 0.013, **p = 0.0072, log-rank tests; ^#^*p* = 0.023, Chi square test.

We evaluated whether low FoxP3 mRNA levels expressed in leukocytes early in disease were predictive of a worse outcome. As shown by Kaplan–Meier curves indicating percent survival of ALS patients to 100 AALS points over time, 66% of patients with FoxP3 levels below the ROC cutoff were above 100 AALS points at the end of the 3.5 years, while only 36.7% of patients with FoxP3 levels above the ROC cutoff were above 100 AALS points (*p* = 0.0072; Fig 9A). In addition, the numbers of deceased ALS patients or patients placed on a ventilator during the 3.5-year evaluation period were compared between patients expressing high *versus* low FoxP3 levels, as shown by Kaplan–Meier curves. The survival data indicate that 35% of patients with FoxP3 levels below the ROC analysis cutoff were placed on a ventilator or were deceased at the end of the 3.5 years, while only 13% of patients with FoxP3 levels above the cutoff were on a ventilator or deceased (*p* = 0.013; Fig 9B). The survival data was also analyzed by the Chi square test, which again revealed that approximately three times more ALS patients with low FoxP3 mRNA levels were placed on a ventilator or were deceased at the end of the 3.5 years compared with ALS patients with high FoxP3 mRNA levels (*p* = 0.023; Fig 9C).

## DISCUSSION

ALS is a heterogeneous disorder with differing rates of progression and lengths of disease. While some ALS patients progress rapidly surviving only a year or two after diagnosis, other patients are slowly progressing and survive 4–6 years or more after diagnosis with a high quality of life for most of the disease. The present study provides evidence of altered Treg numbers and FoxP3 mRNA expression in the peripheral blood of rapidly progressing ALS patients. Leukocytes obtained from patients at all stages of disease contained reduced numbers of CD4^+^CD25^+^ Tregs in rapidly progressing patients, the numbers inversely correlated with progression rates. A trend toward reduced CD4^+^FoxP3^+^ Treg numbers was also observed in leukocytes from rapidly progressing patients. In addition to Treg numbers, the CD4^+^FoxP3^+^ Tregs in rapidly progressing patients contained reduced FoxP3 expression, as measured by FoxP3 fluorescence intensity. As an independent measure, qRT-PCR demonstrated that leukocyte FoxP3 and CD25 mRNA levels were reduced in rapidly progressing patients and inversely correlated with disease progression rates. FoxP3 and CD25 mRNA levels directly correlated with each other; the lower the FoxP3 and CD25 mRNA levels, the more rapid the rate of progression. As Foxp3 is a master transcriptional control gene for Treg-cell development and function, high FoxP3 expression in Tregs is required for their suppressive function, while loss of FoxP3 expression reduces the ability of Tregs to suppress inflammation (Sakaguchi et al, 2010). Therefore, the reduction of FoxP3 expression in Tregs observed in rapidly progressing patients may have diminished the Tregs' suppressive function.

qRT-PCR was also used to examine leukocyte mRNA expression levels of the Th2 transcription factor Gata3, the anti-inflammatory cytokines TGF-β, IL4 and IL10, the Th1 transcription factor Tbx21 and the pro-inflammatory cytokine IFN-γ. As with FoxP3 and CD25, Gata3, TGF-β and IL4 levels were reduced in rapidly progressing patients and inversely correlated with their disease progression rates. Gata3, TGF-β and IL4 mRNA expression levels directly correlated with CD25 and FoxP3 expressions; results suggesting that Tregs in ALS patients could be influencing Th2 leukocytes or *vice versa*. No differences in Tbx21 or IFN-γ expression levels were observed, indicating that not all mRNA levels were depressed. *In vitro*, Th2 lymphocytes and their cytokines TGF, IL4 and IL10 can suppress neuroinflammation, and IL4 can directly suppress microglial neurotoxicity without cell contact (Zhao et al, 2006). Importantly, Th2 lymphocytes are present and appear to suppress inflammation in the cervical cord of the mSOD1 mouse (Beers et al, 2011b). Therefore, a reduction in Th2 lymphocytes in rapidly progressing patients could be contributing to the increased progression rate.

In post-mortem spinal cord tissues, FoxP3 expression was reduced in patients who had progressed rapidly, and Gata3 expression was upregulated in spinal cord tissue from patients who had progressed slowly. Some have suggested that the reduction of FoxP3 and Gata3 mRNA levels in peripheral leukocytes of rapidly progressing patients might be due to an enhanced infiltration of Tregs and Th2 lymphocytes into the CNS compared with the slowly progressing patients. The results of this study suggest the opposite, that if anything, there may be a trend toward enhanced infiltration of Tregs and Th2 lymphocytes into the CNS of slowly progressing patients compared with rapidly progressing patients. Additionally, Tbx21 levels were upregulated to a similar extent in spinal cord tissue from both rapidly and slowly progressing groups of ALS patients *versus* controls, while IFN-γ and NOX2 levels were upregulated only in patients who had progressed rapidly. These results are similar to results observed in the mSOD1 mouse (Beers et al, 2011a) and indicate that Tregs and Th2 lymphocytes could be suppressing inflammation in the spinal cord and influencing the rate at which disease progresses in ALS patients and the mouse model.

The ROC analyses of the original data revealed that FoxP3 and Gata3 expressions accurately reflected the progression rates of ALS patients from all stages of disease at the time of leukocyte collection. FoxP3 and Gata3 expressions were more accurate at reflecting progression rates than the time from first symptom to first exam, a currently used prognostic indicator of future ALS progression rates (Czaplinski et al, 2006; Kollewe et al, 2008). To evaluate whether early FoxP3 or Gata3 expressions could be use to predict future progression rates, leukocytes were obtained from a larger cohort of ALS patients during the early stage of disease and were subsequently followed over a 3.5-year period. FoxP3 levels in leukocytes obtained early in disease accurately reflected their progression rates at the time of collection. Additionally, low FoxP3 levels in leukocytes obtained early in disease accurately predicted future rapid progression rates and importantly, low FoxP3 levels predicted reduced survival; more patients with low FoxP3 levels at the early stages of disease were rapidly progressing at the end of the 3.5-year evaluation period and more were on a ventilator or deceased. Therefore, low FoxP3 mRNA levels were highly accurate predictors of rapid ALS progression rates and reduced survival.

Together, these prospective results suggest that in the ALS pathogenic process Tregs and Th2 lymphocytes slow disease progression rates, and that as the numbers of these cells decline and FoxP3 and Gata3 expressions are reduced, disease progression rates accelerate. This acceleration may be due to the decreased numbers Tregs and Th2 lymphocytes and/or the reduced expression of FoxP3, as high FoxP3 expression is required for the suppressive function of Tregs (Sakaguchi et al, 2010). The mechanism whereby Tregs and Th2 lymphocytes slow ALS progression might partly involve the periphery. However, much of the influence could be due to Tregs and Th2 lymphocytes infiltrating and suppressing inflammation within the CNS in slowly progressing patients. Previously, we had demonstrated enhanced inflammation in the spinal cord of ALS patients, particularly in patients who had been progressing rapidly (Henkel et al, 2004). We and others have demonstrated that inflammation, specifically dendritic and microglial activation, Th1 lymphocytes and pro-inflammatory cytokines, are neurotoxic both *in vitro* and *in vivo*, while Tregs and Th2 lymphocytes, and the TGF-β and IL4 they secrete, can be neuroprotective suppressing the inflammation. The reduced FoxP3 mRNA expression observed in the spinal cord of patients who had been rapidly progressing compared with controls may indicate a diminished influx of Tregs or a down-regulated FoxP3 expression and subsequent loss of function. Additionally, the upregulated Gata3 expression in the spinal cord of slowly progressing patients compared with controls may indicate an enhanced influx of Th2 lymphocytes. In support of Treg and Th2 lymphocytic infiltration, the similarly enhanced Tbx21 expression suggesting increased Th1 lymphocytes in both patients who had progressed rapidly or slowly, yet no upregulated IFN-γ expression in patients who had progressed slowly – as was observed in patients who had progressed rapidly – indicates an active suppression of the Th1 lymphocyte effector functions in slowly progressing patients. This is in contrast to the similar expressions of Tbx21 and IFN-γ in the periphery in both rapidly and slowly progressing patients. Combined, these results suggest that Tregs and Th2 lymphocytes could be infiltrating the spinal cord, influencing both the inflammation in the spinal cord and the rate at which ALS progresses.

These results are similar to those we observed in ALS mice. Previously, we indicated that Tregs may be providing neuroprotective support in the lumbar spinal cord in ALS mice (Beers et al, 2011a), whereas in the cervical spinal cord Th2 cells may also be providing neuroprotective support (Beers et al, 2011b); indicating that both Tregs and Th2 lymphocytes could assist in suppressing the inflammatory processes occurring in these spinal cord regions and modifying disease progression. In addition, an association was demonstrated between the numbers of Tregs and FoxP3 expression with disease progression rate in the mouse model (Beers et al, 2011a). During the early slow phase, Tregs numbers in the periphery were enhanced; conversely, as progression rate accelerated, their numbers declined. Passive transfer of T-lymphocytes and Tregs obtained from donor ALS mice during the early slow phase or *ex vivo* expanded Tregs directly extended this phase and prolonged survival of recipient ALS mice (Banerjee et al, 2008; Beers et al, 2011a). The enhanced neuroprotection derived from slow phase Tregs was attributed to the release of cytokines and subsequent maintenance of M2 microglia and suppression of Teffs. As disease progressed, there was a conversion from supportive Tregs/M2 to injurious Th1/M1 cells. The fact that these T-lymphocyte and spinal cord alterations observed in ALS patients resemble those noted in ALS mice suggests that Tregs and Th2 lymphocytes might also be suppressing the innate and adaptive neurotoxic inflammation in slowly progressing ALS patients and not in rapidly progressing patients. Thus, these results not only indicate that Treg-induced immune suppression may contribute to a slower rate of disease progression in ALS patients, validating the use of mSOD1 mice as a model of immune alterations in ALS, but also suggests that enhancing the numbers of Tregs and Th2 lymphocytes, and/or their anti-inflammatory suppressive functions, might prolong or stabilize their early slow progression rates.

**The paper explained**

PROBLEM:

Amyotrophic lateral sclerosis is a heterogeneous disorder with differing sites of onset, rates of progression, length of disease and pathological mechanisms. An understanding of factors dictating rates of progression would be invaluable in decisions regarding treatment options or clinical therapeutic trials. In mSOD1 transgenic mice, regulatory T-lymphocytes (Tregs) have a documented neuroprotective role slowing disease progression. A key question is whether Tregs have a similar role in influencing rates of progression in patients with ALS.

RESULTS:

Our data indicate that Tregs influence progression rates of ALS patients. The expression of FoxP3, a critical transcription factor required for Treg function, and Gata3, a transcription factor for Th2 lymphocytes, are reduced rapidly progressing patients. FoxP3 and Gata3 expression accurately reflects the patient's current progression rate and present status. Importantly, low FoxP3 expression predicts a future rapid ALS progression rate and reduced survival. Low FoxP3 expression is a more accurate prognostic indicator than the time between first symptom to first exam, allowing improved identification of future rapidly progressing patients.

IMPACT:

The beneficial effect of enhancing Tregs in ALS mice and the association of reduced Treg with faster disease progression and decreased survival in ALS patients provide an important rationale for enhancing Treg populations as a therapeutic option. Enhancing the number of Tregs and their anti-inflammatory suppressive functions in ALS patients could have important therapeutic benefits in slowing the rate of disease progression and stabilizing ALS patients for longer periods of time than other currently available therapies. Additionally, having insight into the clinical course of a patient's disease will assist in patient management and in clinical trial development. On a broader scale, these results may be directly relevant for other neurodegenerative diseases, such as Alzheimer's and Parkinson's diseases.

In a recent report, Rentzos et al. examined Tregs in ALS patients and found that Tregs were significantly reduced compared with normal controls and negatively correlated with disease progression (Rentzos et al, 2011). Mantovani et al. also examined CD4^+^CD25^+^ Tregs in ALS patients and reported that CD4^+^CD25^+^ Tregs were reduced in patients at a less severe stage of disease (Mantovani et al, 2009). While all three reports agree on the reduced Tregs present in ALS patients, our results did not support Mantovani's et al. findings of Tregs being reduced in patients early in disease. Although not significant, our data indicated that the numbers of CD4^+^CD25^High^ Tregs and FoxP3 mRNA expression levels tended to be lower in patients at later stages of disease. Reduced numbers of Tregs have also been observed in other neurodegenerative diseases. Recent studies described reduced numbers of Tregs in patients with Alzheimer's disease, and the more severe the dementia, the greater the decline of Treg numbers (Larbi et al, 2009; Saresella et al, 2010). Additionally, percentages of Treg in Parkinson's disease patients negatively correlated with disease severity (Gendelman; personal communication). These reports, in combination with ours, demonstrate the influence Tregs have on neurodegenerative diseases.

This prospective study indicates that Tregs and Th2 lymphocytes influence ALS progression rates. FoxP3 and Gata3 mRNA levels in leukocytes accurately reflect an ALS patient's current progression rate and could be used to monitor disease progression. Importantly, it suggests that low FoxP3 mRNA levels in leukocyte obtained early in disease predict a rapid progression rate and reduced survival. Having insights into the clinical course of a patient's disease will assist in patient enrollment for future clinical trials and deciding between potential treatment options.

## MATERIALS AND METHODS

### Participants

After receiving IRB approval and written informed consent from patients diagnosed with definite or probable sporadic ALS according to revised El Escorial criteria of the World Federation of Neurology (Brooks, 1994), peripheral blood was drawn and processed for flow analysis or qRT-PCR or postmortem autopsy tissue was obtained and processed for qRT-PCR. Patients were seen at the MDA/ALS Clinic at the Methodist Neurological Institute (Houston, TX) from 2000 to present and evaluated for functional status using the Appel ALS (AALS) score (range: 30–164) (Haverkamp et al, 1995). None of the patients had ongoing infectious diseases. Clinical disease was characterized as early (AALS score: 30–100) or late (AALS score: 101–164). Rate was determined as the change in AALS score, change in time, comparing the initial evaluation with the evaluation at the time of collection or with the evaluation at the end of analysis. Rapidly progressing patients were defined as those progressing at a rate of greater than or equal to 1.5 AALS points/month and slowly progressing patients were those progressing at a rate of less than 1.5 AALS points/month at the time of collection or at the end of the evaluation period. Peripheral blood samples were also donated by healthy control volunteers after giving written informed consent. None of the control volunteers had a history of infectious diseases or other disorders. Three groups of patient samples were used. The first patient group consisted of 54 patients who were from all stages of disease (31 males, 23 females; ages: 35–74 years, mean age: 59 ± 10 SD; 28% of patients had bulbar onset, 72% patients had limb onset) and 33 healthy control volunteers (13 males, 20 females; ages: 31–73 years, mean age: 54 ± 11 SD). A single leukocyte sample collected from each ALS patients and controls were analyzed using flow cytometry, qRT-PCR and ROC analyses. To verify the predictability of FoxP3 expressions, analyses were performed on a second group of patients: a single leukocyte sample from 28 control volunteers (11 males, 17 females; ages: 30–73 years, mean age: 52 ± 12 SD) and 102 ALS patients (69 males, 33 females; ages: 35–82 years, mean age: 59 ± 11 SD; 23% of patients had bulbar onset, 77% patients had limb onset), were collected over a 3-year period, all during the early stages of disease (within a year of their first visit and/or with an AALS score below 100) and compared with progression, which was evaluated every 3 months over a 3.5-year period. The predictability of FoxP3 expressions were evaluated with this second cohort using the optimum ROC cutoff determined with the initial 54 patients' data set (ROC cutoff: FoxP3 = 0.66). qRT-PCR was also performed on postmortem lumbar spinal cord samples from third group of 34 ALS patients (22 males, 12 females; ages: 31–79 years, mean age: 58 ± 10 SD; 28% of patients had bulbar onset, 72% patients had limb onset) and 14 disease controls (8 males, 6 females; ages: 38–76 years, mean age: 57 ± 14 SD).

### Flow cytometry

Blood was processed by The Methodist Hospital Flow Cytometry Lab. Briefly, 100 µl of freshly isolated heparinized (EDTA) peripheral blood was incubated with CD3-PE-Cy7, CD4-AmCyan, CD8-APC-H7, CD25-FITC (BD Biosciences) in the dark for 20 min. Red blood cells (RBC) were lysed and wash with 2 ml of stain buffer (BD Pharmingen). The remaining cells were permeablized and stained with FoxP3-PE according to the manufacturer's instructions (BD Biosciences). The cells were fixed and immediately analyzed using an LSR II™ 8 colour flow cytometer configured with 488, 633, 405 nm lasers. Dead cells were excluded by forward/side scatter gating. CD4^+^CD25^High^ were set based on the control population for each run.

### qRT-PCR of leukocytes from both sets of participants and of postmortem spinal cord tissue

The RBC in the patients' blood were lysed utilizing BD PhamLyse (BD Pharmingen) according to the manufacturer's recommendations. Leukocytes in the blood samples and spinal cord tissues were processed for RNA; RNA isolation and qRT-PCR analyses were performed as described (Henkel et al, 2006). Briefly, RNA was isolated using Trizol (Gibco) and purified using RNeasy (Qiagen) both according to the manufacturers' instructions. The concentrations were determined spectrophotometrically (Thermo Scientific NanoDrop 1000). The qRT-PCR was performed on 10 ng of mRNA using an iQ5 Multicolor Real time PCR Detection System (BioRad), all normalized with β-actin. The primers were created by SigmaGenosys based on Primerbank sequences. The iScript One-step RT-PCR kit with SYBR Green (BioRad) was used to perform qRT-PCR according to the manufacturer's recommendations. Primer efficiencies were assessed by analyzing dilution series of RNAs. The relative expression levels of each mRNAs were calculated using the ΔΔ*C*_t_ method normalizing to β-actin relative to the control samples. The presence of one product of the correct size was verified by gel electrophoresis and melting curve analyses.

### Statistical analyses

Data were analyzed by two-tailed Student's *t*-test (Excel software), linear regression (SigmaStat software), ROC curve analysis (NCSS software), log-rank test (NCSS software), or Chi square (SigmaStat software); *p* < 0.05 was considered statistically significant. Grouped data are expressed as means ± S.E. or expressed as box plots with medians and means denoted. The Center for Biostatistics at The Methodist Hospital Research Institute performed some of the sensitivity/specificity determinations using VassarStats software, and ROC curve analysis using STATA software, version 10.

## For more information

Below is a list relevant web links for additional information on the ALS disease, current ALS research, ALS patient associations and our MDA/ALS Clinic.

http://als.mda.org/

http://www.alsmndalliance.org/

http://www.als.net/

http://www.alsa.org/

http://www.methodisthealth.com/nineurology.cfm?id=34923

## Supplementary material

Detailed facts of importance to specialist readers are published as ”Supporting Information”. Such documents are peer-reviewed, but not copy-edited or typeset. They are made available as submitted by the authors.
