# Regulatory T-lymphocytes mediate amyotrophic lateral sclerosis progression and survival

**DOI:** 10.1002/emmm.201390002

**Published:** 2013-02-04

**Authors:** Jenny S Henkel, David R Beers, Shixiang Wen, Andreana L Rivera, Karen M Toennis, Joan E Appel, Weihua Zhao, Dan H Moore, Suzanne Z Powell, Stanley H Appel

**Erratum to** Jenny S. Henkel, David R. Beers, Shixiang Wen, Andreana L. Rivera, Karen M. Toennis, Joan E. Appel, Weihua Zhao, Dan H. Moore, Suzanne Z. Powell and Stanley H. Appel (2013) Regulatory T-lymphocytes mediate amyotrophic lateral sclerosis progression and survival. EMBO Mol Med 5 (1) DOI: 10.1002/emmm.201201544

The phrase “change in AALS score ÷ change in time” appeared as “change in AALS score, change in time”. This error occurred on pages 2, 10 and 14. The correct sentences are printed below.

Page 2

However, differences were noted when the patients were separated and grouped based on their rate of disease progression at the time of blood collection [rate was determined as the change in AALS score ÷ change in time (months), comparing the initial clinical evaluation with the evaluation performed at the time of blood collection].

Page 10

To evaluate whether early FoxP3 levels could be used to predict future progression rates, the mRNA levels in leukocytes from the 102 patients obtained early in disease were compared with their rate of progression at the end of the evaluation period [rate was determined as the change in AALS score ÷ change in time (months), comparing the initial evaluation with the evaluation at the end of the 3.5-year follow-up period].

Page 14

Rate was determined as the change in AALS score ÷ change in time, comparing the initial evaluation with the evaluation at the time of collection or with the evaluation at the end of analysis.

