# Supplementary material for: Regulatory T-lymphocytes mediate amyotrophic lateral sclerosis progression and survival
Source: EMBO Mol Med. 2012 Nov 9;5(1):64–79. doi: 10.1002/emmm.201201544 (PMC3569654; doi:10.1002/emmm.201201544)
Supplement: Supplementary file 1 [file emmm0005-0064-SD1.pdf]

Manuscript EMM-2012-01544

Regulatory T-lymphocytes Mediate Amyotrophic Lateral Sclerosis Progression and Survival

Jenny S. Henkel, David R. Beers, Shixiang Wen, Andreana L. Rivera, Karen M. Toennis, Joan E. Appel, Weihua Zhao, MD, Dan H. Moore, Suzanne Z. Powell and Stanley H. Appel

Corresponding author: Stanley Appel, Methodist Neurological Institute

Review timeline:

Submission date:	05 May 2012
Editorial Decision:	25 June 2012
Revision received:	22 August 2012
Accepted:	27 September 2012
Author Correspondence:	28 September 2012
Editorial Correspondence:	28 September 2012

Transaction Report:

(Note: With the exception of the correction of typographical or spelling errors that could be a source of ambiguity, letters and reports are not edited. The original formatting of letters and referee reports may not be reflected in this compilation.)

1st Editorial Decision 25 June 2012

Thank you for the submission of your manuscript "Regulatory T-lymphocytes Mediate Amyotrophic Lateral Sclerosis Progression and Survival" to EMBO Molecular Medicine and please accept my sincere apologies for the delayed reply. We have now finally heard back from the three referees whom we asked to evaluate your manuscript. You will see that they find the topic of your manuscript potentially interesting and are supportive of your study. However, one reviewer also raises significant concerns on the study, which should be addressed in a major revision of the manuscript.

In particular, reviewer #3 highlights that the data regarding FoxP3, CD25 and Gata3 levels in spinal cord tissue should be strengthened and that clinical data on the potential infection status of the patients should be included. In addition, this reviewer highlights that the manuscript text should be edited to make it better accessible for a general audience.

Given the balance of these evaluations, we feel that we can consider a revision of your manuscript if you can convincingly address the issues that have been raised within the space and time constraints outlined below.

Revised manuscripts should be submitted within three months of a request for revision. They will otherwise be treated as new submissions, unless arranged differently with the editor.

© EMBO

I look forward to seeing a revised form of your manuscript as soon as possible.

Yours sincerely,

Editor
EMBO Molecular Medicine

***** Reviewer's comments *****

Referee #1 (Comments on Novelty/Model System):

The findings that Tregs can influence ALS progression rates and that low FoxP3 is predictive of fast ALS progression are novel. The study was carried out with relatively arge cohorts of human ALS and controls. The results have medical impact for therapeutic development and for the use in clinical trials of novel markers to identify rapidly progressing ALS patients.

Referee #1 (Other Remarks):

The authors have analyzed by flow cytometry T lymphocytes from 54 ALS patients in disease progression. The number of CD4+ CD25 high Tregs and intensity of CD4+FoxP3 Tregs were found to be reduced in rapidly progressing ALS. The mRNA levels for FoxP3, CD25, TGFbeta, IL4 and Gata3 were also reduced in rapidly progressing ALS patients when compared with slowly progressing patients. On the other hand, the levels of IL10, IFNgamma and Tbx21 were similar in slow and rapid ALS. The levels of FoxP3 mRNA in leukocytes were evaluated in another cohort of 102 ALS patients during a period of 3.5 years. Low FoxP3 was found to be predictive of rapid ALS progression. From these results it is proposed that a FoxP3 reduction may diminish the suppressive function of Tregs.
In mutant SOD1 mice, Tregs were found previously to play neuroprotective function. The data presented in this study further demonstrate that Tregs can influence ALS progression rates in human ALS. The findings might have implications for therapeutic development and also suggest the use of novel markers to identify rapidly progressing ALS patients. The results are novel and support the conclusions. The paper merits publication in its present form.
Minor Point:
Fig 1D there is a typing mistake: Progression

Referee #2:

This is a carefully done study that links regulatory T cells and Th2 type responses to disease course in ALS. It clearly demonstrates the importance of the immune system in this disease, even though the damage is to motor neurons. The authors should make clear where in the pathway of disease they believs the immune system is specifically acting.

Referee #3 (Comments on Novelty/Model System):

Humans really are a good model for human disease.

Referee #3 (Other Remarks):

Over the past five years, the Appel group has investigated the role of T lymphocytes in the progression of ALS-like disease in mutant SOD1 mice that develop fatal paralysis. They have established that two subpopulations of T cells, namely CD4+ T helper (Th) and regulatory T (Treg) cells ameliorate disease progression in this mouse model. In particular Tregs, whose role is to suppress immune responses that may be harmful to the host, are upregulated during the stable phase of ALS-like disease in SOD1 mice, suggesting that they may be involved in slowing disease progression.

In the current clinical study, Henkel and colleagues test this hypothesis by investigating the levels of regulatory T cells or Tregs in peripheral blood of 54 ALS patients and 33 healthy control volunteers of approximately the same age range. They report that the levels of Tregs in peripheral blood of rapidly progressing ALS are significantly reduced, compared to controls and patients with slowly progressing ALS. They go on to measure the mRNA levels of the Treg marker FoxP3 in peripheral leukocytes from the same patients and they find the same correlation: ALS patients with rapid progression have significantly lower mRNA levels of this marker compared to the other groups. The authors show that the anti-inflammatory markers IL4, TGF-beta and the T-helper transcription factor Gata3 are also reduced in the rapidly progressing group.

Most importantly, Henkel and colleagues show that the levels of FoxP3 in peripheral lymphocytes is a better predictor of the rate of disease progression than the current measures, which are based on the time between occurrence of the first symptom and the first clinic visit. To prove the predictive value of FoxP3 levels for future rates of disease progression, the investigators followed a 3.5-year prospective study of 102 ALS patients and 28 control volunteers. This analysis convincingly shows that low FoxP3 levels are predictive for future rapid disease progression, albeit high FoxP3 levels don't always indicate slowly progressing disease.

This is a very important study for clinical practice, as leukocyte FoxP3 levels could help monitor the efficiency of current and future treatments used in ALS patients. I would strongly recommend the publication of the work in EMBO Molecular Medicine, upon revisions detailed below.

One key aspect - and I can't stress it too much - is that to reach a broad audience effectively the text needs substantial attention to make it readable by a general audience. Beginning with the Summary, repetition should be removed. Added to this, the immunologic-speak (the current text is replete with avoidable jargon) could be more effectively suppressed so that many more investigators will be able to follow the detailed text.

Points of criticism

1. In my view, the weakest part of the manuscript is the analysis on spinal cord tissues shown in Figure 6. The authors claim that the spinal cord changes reflect those seen in peripheral leukocytes of the same patients, yet I do not see this in Figure 6. In contrast, while FoxP3, CD25 and Gata3 levels are both clearly reduced in peripheral leukocytes of the rapidly progressing group, in the spinal cord tissues the differences are much less obvious. FoxP3 is barely reduced in the rapidly progressing group and CD25 levels seem equal between the slow and rapid groups. Gata3 seems rather increased in both ALS groups compared to controls. I am wondering if that discrepancy may be due to the smaller number of autopsy samples used compared to the blood samples - there were only 34 ALS and 14 control spinal cords, compared to 54 ALS and 33 control blood samples respectively. Also, I was not able to find in the manuscript the exact number of rapid ALS patients that were included in the 34 spinal cord set - this should be clearly indicated in the manuscript.

2. I think it is crucial to settle the above point in order to support the authors' hypothesis that the differences found in peripheral leukocytes reflect the level of T-cell infiltration within the spinal cord - a hypothesis that I find very intriguing and one that would strongly support the beneficial effects of Tregs and T helper cells in human ALS. In order to do this, the authors should check for infiltration of these two T-cell subpopulations in the spinal cords of their cohort of patients by immunohistochemistry and report whether the level of Tregs and T helper cells inversely correlate with ALS disease progression - as the peripheral blood analysis would suggest.

3. The Gata3 reduction in peripheral leukocytes of the first cohort of patients seems more profound than that of FoxP3. Yet the authors focus their later prospective analysis study on FoxP3. I think they should do the same analysis shown in Figures 8 and 9 (the prospective ALS patient cohort) for Gata3 as well. Based on what is shown on Figure 4A, I expect the correlation and prediction value of Gata3 mRNA levels to be even better than that of FoxP3.

4. The current text left me wondering if the authors have any clinical data on the potential infection status of the ALS patients used in the study. I think that it would be important to exclude significant differences of the infection status of the different ALS groups, as these could account for the changes in the levels of T-cells described here. This analysis would be particularly important if the

authors are not able to prove a correlation with the levels of T-cell infiltration in the spinal cord samples.

5. I was wondering how accurate the definition of rapid versus slow progression of disease is in ALS patients. I think that the general audience of EMBO Molecular Medicine would benefit from a more elaborate explanation/clarification of this point.

Minor point.

6. There is a typo in Figure 1D at the title of the x-axis. It should be "PROGRESSION RATES" instead of "PROGRSSION RATES".

1st Revision - Authors' Response 22 August 2012

The authors would like to thank you and each referee for their comments/suggestions. We appreciate their input and would like to have the opportunity to address each of their comments. We have included all three referee's entire reviews and responded in order to each comment/criticism. In the text that follows, each referee's comments are in italics and are followed by our response.

Referee 1 begins by stating that *"The findings that Tregs can influence ALS progression rates and that low FoxP3 is predictive of fast ALS progression are novel. The study was carried out with relatively large cohorts of human ALS and controls. The results have medical impact for therapeutic development and for the use in clinical trials of novel markers to identify rapidly progressing ALS patients."*

Referee 1 continues and states that "*The authors have analysed by flow cytometry T lymphocytes from 54 ALS patients in disease progression. The number of CD4+ CD25 high Tregs and intensity of CD4+FoxP3 Tregs were found to be reduced in rapidly progressing ALS. The mRNA levels for FoxP3, CD25, TGFbeta, IL4 and Gata3 were also reduced in rapidly progressing ALS patients when compared with slowly progressing patients. On the other hand, the levels of IL10, IFNgamma and Tbx21 were similar in slow and rapid ALS. The levels of FoxP3 mRNA in leukocytes were evaluated in another cohort of 102 ALS patients during a period of 3.5 years. Low FoxP3 was found to be predictive of rapid ALS progression. From these results it is proposed that a FoxP3 reduction may diminish the suppressive function of Tregs.*

In mutant SOD1 mice, Tregs were found previously to play neuroprotective function. The data presented in this study further demonstrate that Tregs can influence ALS progression rates in human ALS. The findings might have implications for therapeutic development and also suggest the use of novel markers to identify rapidly progressing ALS patients. The results are novel and support the conclusions. The paper merits publication in its present form."

Our response:

We thank referee 1 for his/her comments.

Referee 1 also indicates that in "*The Fig 1D there is a typing mistake: Progression.*"

Our response:

We thank the referee for pointing out this typo and have made the indicated correction.

Referee 2 states that "*This is a carefully done study that links regulatory T cells and Th2 type responses to disease course in ALS. It clearly demonstrates the importance of the immune system in this disease, even though the damage is to motor neurons. The authors should make clear where in the pathway of disease they believe the immune system is specifically acting.*"

Our response:

We also thank referee 2 for his/her comments and have included the following paragraph in the Discussion section.

"Together, these prospective results suggest that in the ALS pathogenic process Tregs and Th2 lymphocytes slow disease progression rates, and that as the numbers of these cells decline and FoxP3 and Gata3 expressions are reduced, disease progression rates accelerate. This acceleration may be due to the decreased numbers Tregs and Th2 lymphocytes and/or the reduced expression of FoxP3, as high FoxP3 expression is required for the suppressive function of Tregs (Sakaguchi et al., 2010). The mechanism whereby Tregs and Th2 lymphocytes slow ALS progression might partly involve the periphery. However, much of the influence could be due to Tregs and Th2 lymphocytes infiltrating and suppressing inflammation within the CNS in slowly progressing patients. Previously, we had demonstrated enhanced inflammation in the spinal cord of ALS patients, particularly in patients who had been progressing rapidly (Henkel et al, 2004). We and others have demonstrated that inflammation, specifically dendritic and microglial activation, Th1 lymphocytes, and pro-inflammatory cytokines, are neurotoxic both in vitro and in vivo, while Tregs and Th2 lymphocytes, and the TGFβ and IL4 they secrete, can be neuroprotective suppressing the inflammation. The reduced FoxP3 mRNA expression observed in the spinal cord of patients who had been rapidly progressing compared with controls may indicate a diminished influx of Tregs or a down-regulated FoxP3 expression and subsequent loss of function. Additionally, the up regulated Gata3 expression in the spinal cord of slowly progressing patients compared with controls may indicate an enhanced influx of Th2 lymphocytes. In support of Treg and Th2 lymphocytic infiltration, the similarly enhanced Tbx21 expression suggesting increased Th1 lymphocytes in both patients who had progressed rapidly or slowly, yet no up regulated IFNγ expression in patients who had progressed slowly - as was observed in patients who had progressed rapidly - indicates an active suppression of the Th1 lymphocyte effector functions in slowly progressing patients. This is in contrast to the similar expressions of Tbx21 and IFNγ in the periphery in both rapidly and slowly progressing patients. Combined, these results suggest that Tregs and Th2 lymphocytes could be infiltrating the spinal cord, influencing both the inflammation in the spinal cord and the rate at which ALS progresses."

Referee 3 begins by stating that "*Over the past five years, the Appel group has investigated the role of T lymphocytes in the progression of ALS-like disease in mutant SOD1 mice that develop fatal paralysis. They have established that two subpopulations of T cells, namely CD4+ T helper (Th) and regulatory T (Treg) cells ameliorate disease progression in this mouse model. In particular Tregs, whose role is to suppress immune responses that may be harmful to the host, are up regulated during the stable phase of ALS-like disease in SOD1 mice, suggesting that they may be involved in slowing disease progression.*

In the current clinical study, Henkel and colleagues test this hypothesis by investigating the levels of regulatory T cells or Tregs in peripheral blood of 54 ALS patients and 33 healthy control volunteers of approximately the same age range. They report that the levels of Tregs in peripheral blood of rapidly progressing ALS are significantly reduced, compared to controls and patients with slowly progressing ALS. They go on to measure the mRNA levels of the Treg marker FoxP3 in peripheral leukocytes from the same patients and they find the same correlation: ALS patients with rapid progression have significantly lower mRNA levels of this marker compared to the other groups. The authors show that the anti-inflammatory markers IL4, TGF-beta and the T-helper transcription factor Gata3 are also reduced in the rapidly progressing group.

Most importantly, Henkel and colleagues show that the levels of FoxP3 in peripheral lymphocytes is a better predictor of the rate of disease progression than the current measures, which are based on the time between occurrence of the first symptom and the first clinic visit. To prove the predictive value of FoxP3 levels for future rates of disease progression, the investigators followed a 3.5-year prospective study of 102 ALS patients and 28 control volunteers. This analysis convincingly shows that low FoxP3 levels are predictive for future rapid disease progression, albeit high FoxP3 levels don't always indicate slowly progressing disease.

This is a very important study for clinical practice, as leukocyte FoxP3 levels could help monitor the efficiency of current and future treatments used in ALS patients. I would strongly recommend the publication of the work in EMBO Molecular Medicine, upon revisions detailed below."

Our response:

We thank referee 3 for his/her comments.

Referee 3 continues and indicates that "*One key aspect - and I can't stress it too much - is that to reach a broad audience effectively the text needs substantial attention to make it readable by a general audience. Beginning with the Summary, repetition should be removed. Added to this, the immunologic-speak (the current text is replete with avoidable jargon) could be more effectively suppressed so that many more investigators will be able to follow the detailed text."*

Our response:

We have simplified the "Abstract", removed any flow cytometric jargon, and removed any repetition to read as follows:

"In amyotrophic lateral sclerosis (ALS) mice, regulatory T-lymphocytes (Tregs) are neuroprotective, slowing disease progression. To address whether Tregs and FoxP3, a transcription factor required for Treg function, similarly influence progression rates of ALS patients, T-lymphocytes from patients were assessed by flow cytometry. Both numbers of Tregs and their FoxP3 protein expressions were reduced in rapidly progressing ALS patients and inversely correlated with progression rates. The mRNA levels of FoxP3, TGFβ, IL4, and Gata3, a Th2 transcription factor, were reduced in rapidly progressing patients and inversely correlated with progression rates. Both FoxP3 and Gata3 were accurate indicators of progression rates. No differences in IL10, Tbx21, a Th1 transcription factor, or IFNγ expression were found between slow and rapid patients. A 3.5-year prospective study with a second larger cohort revealed early reduced FoxP3 levels were indicative of progression rates at collection and predictive of future rapidly progressing disease and attenuated survival. Collectively, these data suggest Tregs and Th2 lymphocytes influence disease progression rates. Importantly, early reduced FoxP3 levels could be used to identify rapidly progressing patients."

In "The paper explained" section, we have also simplified the "results" sub-section.

In the main body of the manuscript, under the "Results" section, the first sub-section is the only sub-section where we included flow cytometric jargon. For clarity, in this section we felt it was imperative that that we correctly define the specific type of regulatory T-lymphocytes that we were investigating: $CD4^{+}CD25^{High}$ or $CD4^{+}FoxP3^{+}$. Additionally, we had initially only defined the "disease progression rate" of the ALS patients in the "Patients and Methods" section, but we were asked to also re-define the "rate" in the "Results" section.

In the "Discussion" section, the first paragraph is the only place where we discuss $CD4^{+}CD25^{High}$ or $CD4^{+}FoxP3^{+}$ regulatory T-lymphocyte. Again, we felt that we needed to indicate the type of regulatory T-lymphocyte for clarity and precision.

Referee 3 goes on to state "*Points of criticism*:

1. In my view, the weakest part of the manuscript is the analysis on spinal cord tissues shown in Figure 6. The authors claim that the spinal cord changes reflect those seen in peripheral leukocytes of the same patients, yet I do not see this in Figure 6. In contrast, while FoxP3, CD25 and Gata3 levels are both clearly reduced in peripheral leukocytes of the rapidly progressing group, in the spinal cord tissues the differences are much less obvious. FoxP3 is barely reduced in the rapidly progressing group and CD25 levels seem equal between the slow and rapid groups. Gata3 seems rather increased in both ALS groups compared to controls. I am wondering if that discrepancy may be due to the smaller number of autopsy samples used compared to the blood samples - there were only 34 ALS and 14 control spinal cords, compared to 54 ALS and 33 control blood samples respectively. Also, I was not able to find in the manuscript the exact number of rapid ALS patients that were included in the 34 spinal cord set - this should be clearly indicated in the manuscript."

Our response:

We agree with the reviewer that the title of this sub-section was confusing; therefore, we have changed the title of this sub-section to read "*Levels of anti- and pro- inflammatory factors in ALS spinal cord."* Our goals for this sub-section were: 1) to demonstrate that the reduction of peripheral Tregs numbers and FoxP3 and Gata3 expressions in rapidly progressing patients were not due to an enhanced infiltration of Tregs and Th2 lymphocytes into the CNS and 2) to find evidence that Tregs and Th2 lymphocytes might be suppressing inflammation in slowly progressing patients. Toward the first goal, we demonstrated that FoxP3 and Gata3 expressions in the CNS were not enhanced in rapidly progressing patients. If anything, the trend was for the opposite: FoxP3 and Gata3 mRNA expressions levels were trending lower in spinal cord tissues of rapidly progressing ALS patient. Toward the second goal, we found equivalent levels of Tbx21 in the CNS in rapidly and slowly progressing patients suggesting similar numbers of Th1 lymphocytes, but no increase in IFNγ in slowly progressing patients - the IFNγ levels were identical to controls. This result suggests an active suppression of Th1 lymphocyte effector functions in slowly progressing patients.

Therefore, in addition to the title change, we have added the following sentence to the "Results" section: "Therefore, while these analyses were performed on tissues from patients after death, the reduced FoxP3 and Gata3 expressions in peripheral leukocytes of patients who had progressed rapidly do not appear to be due to an enhanced influx of Tregs and Th2 lymphocytes into the CNS."

We have also added the following clarification to the third paragraph of the "Discussion" section of the manuscript: "In post-mortem spinal cord tissues, FoxP3 expression was reduced in patients who had progressed rapidly, and Gata3 expression was up regulated in spinal cord tissue from patients who had progressed slowly. Some have suggested that the reduction of FoxP3 and Gata3 mRNA levels in peripheral leukocytes of rapidly progressing patients might be due to an enhanced infiltration of Tregs and Th2 lymphocytes into the CNS compared with the slowly progressing patients. These results suggest the opposite, that if anything, there may be a trend toward enhanced infiltration of Tregs and Th2 lymphocytes into the CNS of slowly progressing patients compared to the rapidly progressing patients."

Finally to address the number of samples in each group, we have added "from 15 patients who had progressed rapidly, 19 patients who had progressed slowly and 14 controls" to the end of the first sentence in this sub-section of the "Results", now entitled *"Levels of anti- and pro- inflammatory factors in ALS spinal cord."*

2. I think it is crucial to settle the above point in order to support the authors' hypothesis that the differences found in peripheral leukocytes reflect the level of T-cell infiltration within the spinal cord - a hypothesis that I find very intriguing and one that would strongly support the beneficial effects of Tregs and T helper cells in human ALS. In order to do this, the authors should check for infiltration of these two T-cell subpopulations in the spinal cords of their cohort of patients by immunohistochemistry and report whether the levels of Tregs and T helper cells inversely correlate with ALS disease progression - as the peripheral blood analysis would suggest.

Our response:

We agree with the referee's suggestion that demonstrating the presence of $FoxP3^+$ Tregs and $Gata3^+$ Th2 lymphocytes in the CNS would enhance our contention that these lymphocytes might be actively suppressing inflammation in the CNS. However, as we stated earlier, the equivalent levels of Tbx21 in rapidly and slowly progressing patients suggesting similar numbers of Th1 lymphocytes, but no increase in IFNγ in slowly progressing patients - the IFNγ levels were identical to controls - suggest an active suppression of Th1 lymphocyte effector functions in slowly progressing patients. Nevertheless, we made every effort to carry out the suggested immunohistochemical evaluations. We first immunohistochemically stained control lymph node tissue for FoxP3, Tbx-21, and Gata3 using the DAB method for detection. We were able to detect all three epitopes in the different sections of lymph nodes in what morphologically appeared to be lymphocytes. However, our clinical neuropathologist warned us prior to staining sections of ALS patient post-mortem spinal cords that the tissues may have been compromised because of the age of the tissue and other variables. Because we were successful in staining the lymphoid tissue, we did try to stain sections from spinal cords of several patients for these three epitopes. The neuropathologist's speculation on the integrity these tissues proved correct. In each case, the background staining was too high with significant non-specific signal. Even after trying to optimize the staining conditions, the background noise and non-specific signal remained unsuitably high. Again, it is not clear if it was the age of the tissue or the staining protocol that were the issues. Thus, we were not able to comply with the referee's suggestion and were unable to identify convincingly positive FoxP3, Tbx-21, and Gata-3 lymphocytes in the spinal cords of ALS patients. However, as the major criticism of this referee were the words we used to describe our results in this section, and as we mentioned earlier have appropriately modified our description, we hope we have addressed his/her concerns..

3. The Gata3 reduction in peripheral leukocytes of the first cohort of patients seems more profound than that of FoxP3. Yet the authors focus their later prospective analysis study on FoxP3. I think they should do the same analysis shown in Figures 8 and 9 (the prospective ALS patient cohort) for Gata3 as well. Based on what is shown on Figure 4A, I expect the correlation and prediction value of Gata3 mRNA levels to be even better than that of FoxP3.

Our response:

We thank the referee for this interesting observation. We performed a ROC analysis on Gata3 levels in leukocytes from patients at all stages of disease and found that Gata3 levels were as indicative of the patients' current progression rates as were FoxP3 levels. However when we examined the levels in leukocytes from patients in the early stages of their disease, most patients expressed high Gata3 levels, limiting the predictive value of Gata3. We have now added these additional results to the text and the figures of our revised manuscript.

4. The current text left me wondering if the authors have any clinical data on the potential infection status of the ALS patients used in the study. I think that it would be important to exclude significant differences of the infection status of the different ALS groups, as these could account for the changes in the levels of T-cells described here. This analysis would be particularly important if the authors are not able to prove a correlation with the levels of T-cell infiltration in the spinal cord samples.

Our response:

This was something that we also had considered; however, none of the patients analysed in our study had an on-going infectious disease. We have included a sentence in the "Patients and Methods" section that reads "None of the patients had on-going infectious diseases." In addition, none of the controls had any infectious diseases which had been previously indicated in the "Patients and Methods" section.

5. I was wondering how accurate the definition of rapid versus slow progression of disease is in ALS patients. I think that the general audience of EMBO Molecular Medicine would benefit from a more elaborate explanation/clarification of this point.

Our response:

When we were deciding on an appropriate delineation of fast versus slow progressing patient, we reviewed our historical records of ALS patient progression rates. A progression rate of 1.5 Appel ALS (AALS) points/month separated the patients surviving only a year or two after diagnosis from those that survive up to 6 years or more after diagnosis and also divided these patients approximately in half; half of the patients were progressing 1.5 AALS points/month or more, while the other half was progressing slower than 1.5 AALS points/month. A statement to this effect has been added to the "Results" section of our manuscript.

The Appel ALS scoring system used in our clinic and in this study has been validated and is described in Haverkamp et al (1995). This reference was indicated in the "Patients and Methods" section but has now been added to the "Results" section of our manuscript as well.

To provide a more general description for a broader audience, we have included the following statement in the "Discussion" section of our revised manuscript: "ALS is a heterogeneous disorder with differing rates of progression and lengths of disease. While some ALS patients progress rapidly surviving only a year or two after diagnosis, other patients are slowly progressing and survive up to 6 years or more after diagnosis with a high quality of life for most of the disease."

Minor point.

6. There is a typo in Figure 1D at the title of the x-axis. It should be "PROGRESSION RATES" instead of "PROGRSSION RATES".

Our response:

As indicated above, we thank the referee for pointing out this typo and have made the indicated correction

Again, we would like to thank you and each referee for their critical review of our manuscript. We believe we have made a concerted effort to fully address each of their concerns/suggestions. As we have communicated in this revised manuscript, our data indicates that low FoxP3 mRNA levels are not only predictive of future rapid progression rates, but predict an attenuated survival for ALS patients. Having these insights into the clinical course of a patient's disease will assist in patient enrolment for future clinical trials and deciding between potential treatment options; these results accentuate the importance of patient selection criteria for any immune cell based therapy. Finally, all authors have read and agreed to the content of this letter and the revised manuscript, and are grateful for the opportunity to submit a revised manuscript to EMBO Molecular Medicine.

2nd Editorial Decision 27 September 2012

Please find enclosed the final reports on your manuscript. We are pleased to inform you that your manuscript is accepted for publication and is now being sent to our publisher to be included in the next available issue of EMBO Molecular Medicine.

Please send me the information queried by the Reviewer below, which we will include into the manuscript for you.

Congratulations on your interesting work,

Editor
EMBO Molecular Medicine

***** Reviewer's comments *****

Referee #3 (Comments on Novelty/Model System):

The model system is Human!

Referee #3 (Remarks):

This is a considerably improved revision of one this referee has previously commented upon. As in the initial review, over the past five years, the Appel group has investigated the role of T lymphocytes in the progression of ALS-like disease in mutant SOD1 mice that develop fatal paralysis. They have established that two subpopulations of T cells, namely CD4+ T helper (Th) and regulatory T (Treg) cells ameliorate disease progression in this mouse model. In particular Tregs, whose role is to suppress immune responses that may be harmful to the host, are upregulated during the stable phase of ALS-like disease in SOD1 mice, suggesting that they may be involved in slowing disease progression.

In the current clinical study, Henkel and colleagues test this hypothesis by investigating the levels of regulatory T cells or Tregs in peripheral blood of 54 ALS patients and 33 healthy control volunteers of approximately the same age range. They report that the levels of Tregs in peripheral blood of rapidly progressing ALS are significantly reduced, compared to controls and patients with slowly progressing ALS. They go on to measure the mRNA levels of the Treg marker FoxP3 in peripheral leukocytes from the same patients and they find the same correlation: ALS patients with rapid progression have significantly lower mRNA levels of this marker compared to the other groups. The authors show that the anti-inflammatory markers IL4, TGF-beta and the T-helper transcription factor Gata3 are also reduced in the rapidly progressing group. Most importantly, Henkel and colleagues show that the levels of FoxP3 in peripheral lymphocytes is a better predictor of the rate of disease progression than the current measures, which are based on the time between occurrence of the first symptom and the first clinic visit. To prove the predictive value of FoxP3 levels for future rates of disease progression, the investigators followed a 3.5-year prospective study of 102 ALS patients and 28 control volunteers. This analysis convincingly shows that low FoxP3 levels are predictive for future rapid disease progression, albeit high FoxP3 levels don't always indicate slowly progressing disease.

This is a very important study for clinical practice, as leukocyte FoxP3 levels could help monitor the efficiency of current and future treatments used in ALS patients. My earler detailed criticisms have largely been addressed satisfactorily. I strongly recommend the publication of the work in EMBO Molecular Medicine.

One final point:

The authors refer at least twice that CD4+ T-lymphocytes "slowed disease progression" in the most widely used SOD1 mutant mouse model. By how much? So many "treatments" in this mouse yield very modest effects - so much so that many just don't believe them. I didn't go look it up, but the authors ought to add more specificity.

Author Correspondence 28 September 2012

Regarding the improvement due to CD4 cells injected into the mSOD1 mouse, we observed a 50% increase in disease duration with CD4+ T cells.

Editorial Correspondence 28 September 2012

Thank you for the additional information, which we added to the manuscript. Please carefully check the proofs for whether you feel they were included in an appropriate place.

Yours sincerely,

Editor
EMBO Molecular Medicine
