# Supplementary material for: Regulatory T-lymphocytes mediate amyotrophic lateral sclerosis progression and survival
Source: EMBO Mol Med. 2012 Nov 9;5(1):64–79. doi: 10.1002/emmm.201201544 (PMC3569654; doi:10.1002/emmm.201201544)
Supplement: Supplementary file 2 [file emmm0005-0064-SD2.pdf]

**Supporting Information**

This Supporting Information formed as part of the original submission and has been peer reviewed.

Supplement to: Henkel JS, Beers DR, Wen S et al. Regulatory T-Lymphocytes Mediate Amyotrophic Lateral Sclerosis Progression and Survival.
EMBO Mol Med; submitted 2012.

**TABLE OF CONTENTS** **Page**


**Supporting Information Figure 1** 2
Leukocyte IL10 mRNA expression levels are not reduced in rapidly progressing ALS patients.

**Supporting Information Figure 2** 3
Leukocyte TGFβ and IL4 mRNA expressions are associated with FoxP3, CD25, and Gata3 mRNA expressions.

**Supporting Information Figure 3** 4
Leukocyte Tbx21 (Tbet) and IFNγ mRNA expression levels are not different in ALS patients regardless of the progression rate.

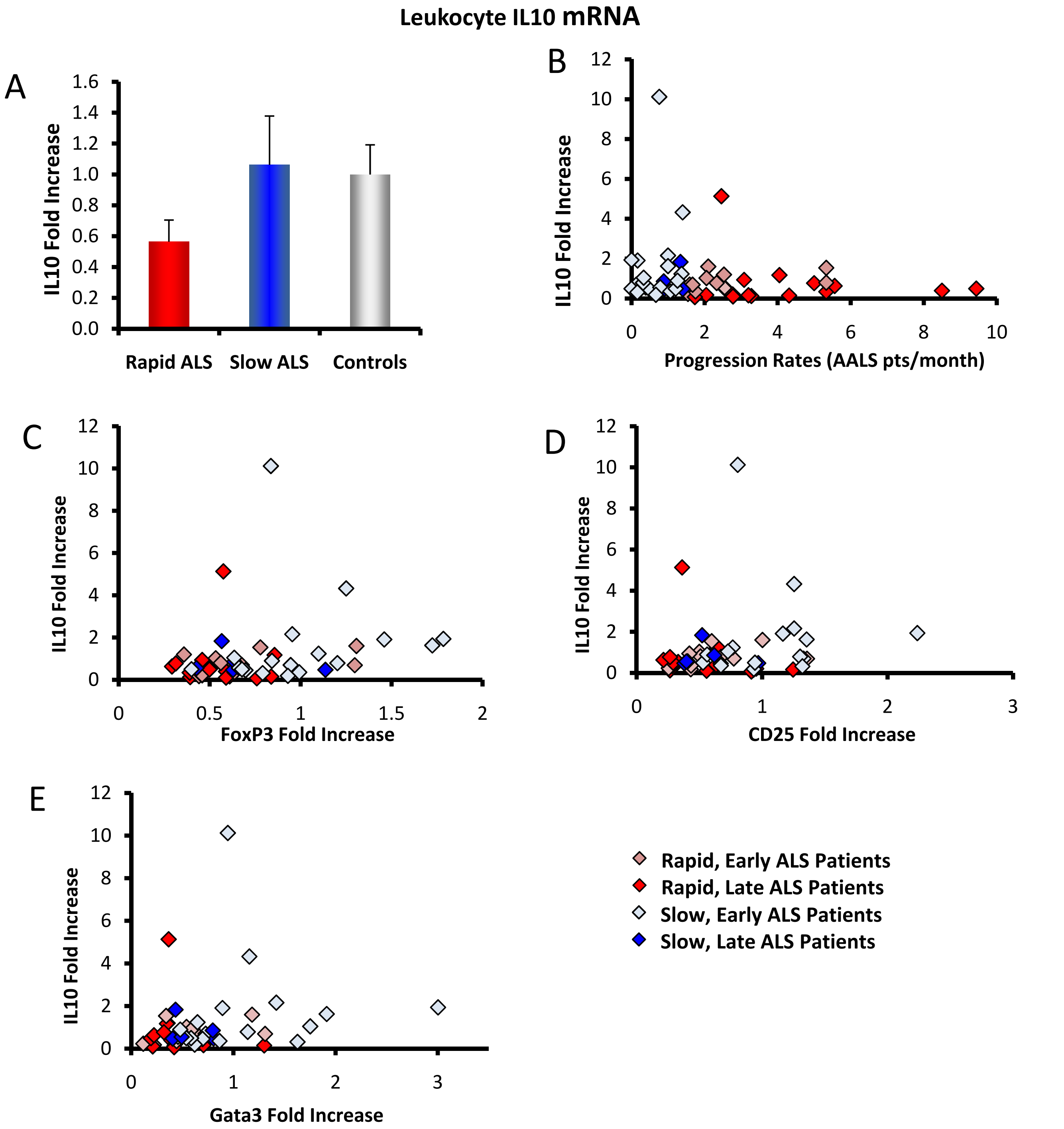


**Supporting Information Figure 1**

Leukocyte IL10 mRNA expression levels are not reduced in rapidly progressing ALS patients. qRT-PCR was utilized to evaluate mRNA expression levels of IL10 in leukocytes obtained from 54 ALS patients through all stages of disease and 33 control volunteers. (A, B) In ALS patients, there was a trend toward reduced IL10 mRNA expression in rapidly progressing ALS patients compared with slowly progressing patients (p=0.161) and compared with controls (p=0.0732; T-test), but no correlation with rate of disease progression (R=0.156, p=0.285; linear regression). There were trends toward correlations between IL10 and (C) FoxP3 or (E) Gata3 mRNA levels (p=0.115, p=0.185, respectively). IL-10 mRNA expression levels in ALS patients did not correlate with (D) CD25 mRNA expression levels (p=0.338; linear regression). Slowly progressing ALS patients = AALS points/month<1.5; rapidly progressing ALS patients = AALS points/month>1.5, at the time of collection. ALS patients early in disease = AALS score<100; ALS patients late in disease = AALS score≥100, at the time of collection.

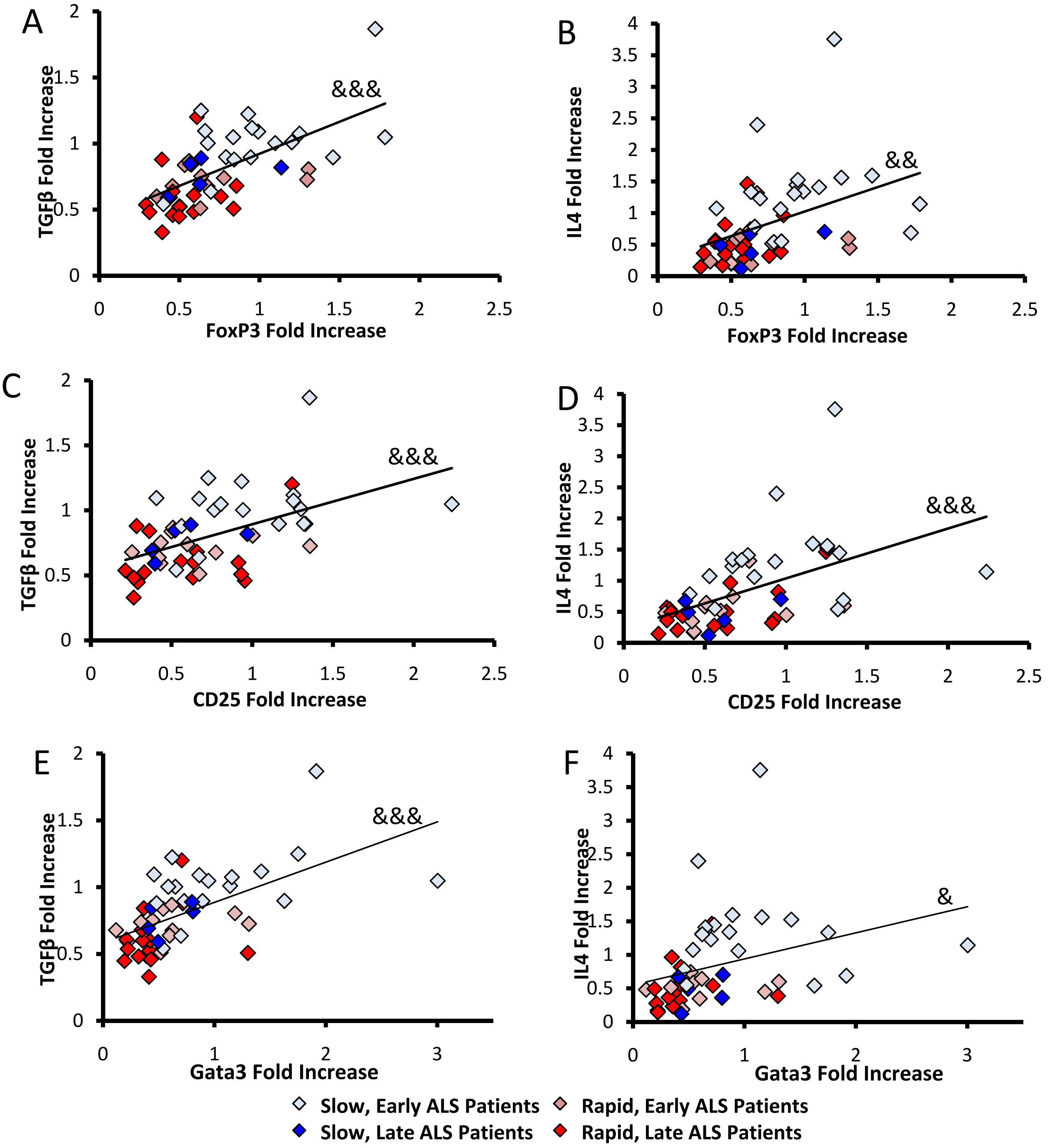


**Supporting Information Figure 2**

Leukocyte TGFβ and IL4 mRNA expressions are associated with FoxP3, CD25, and Gata3 mRNA expressions. The leukocyte TGFβ and IL4 mRNA expression levels were compared with FoxP3, CD25, and Gata3 mRNA expression levels to assess whether they were associated. (A) TGFβ and FoxP3 levels directly correlated (R=0.611; linear regression). (B) IL4 and FoxP3 levels correlated directly (R=0.417; linear regression). (C) TGFβ and CD25 mRNA levels directly correlated (R=0.512; linear regression). (D) IL4 and CD25 mRNA levels correlated directly (R=0.500; linear regression. (E) TGFβ and Gata3 levels correlated (R=0.577; linear regression). (F) IL4 and Gata3 mRNA levels correlated (R=0.317; linear regression). Note that slowly progressing patients early in their disease expressed the highest IL4, TGFβ, FoxP3, CD25, and Gata3 mRNA levels, whereas rapidly progressing patients late in their course of disease expressed the lowest levels of IL4, TGFβ, FoxP3, CD25, and Gata3 mRNAs. $^{\&}$p≤0.027, $^{\&\&}$p≤0.003, $^{\&\&\&}$p≤0.001.

# Supporting Information Figure 3

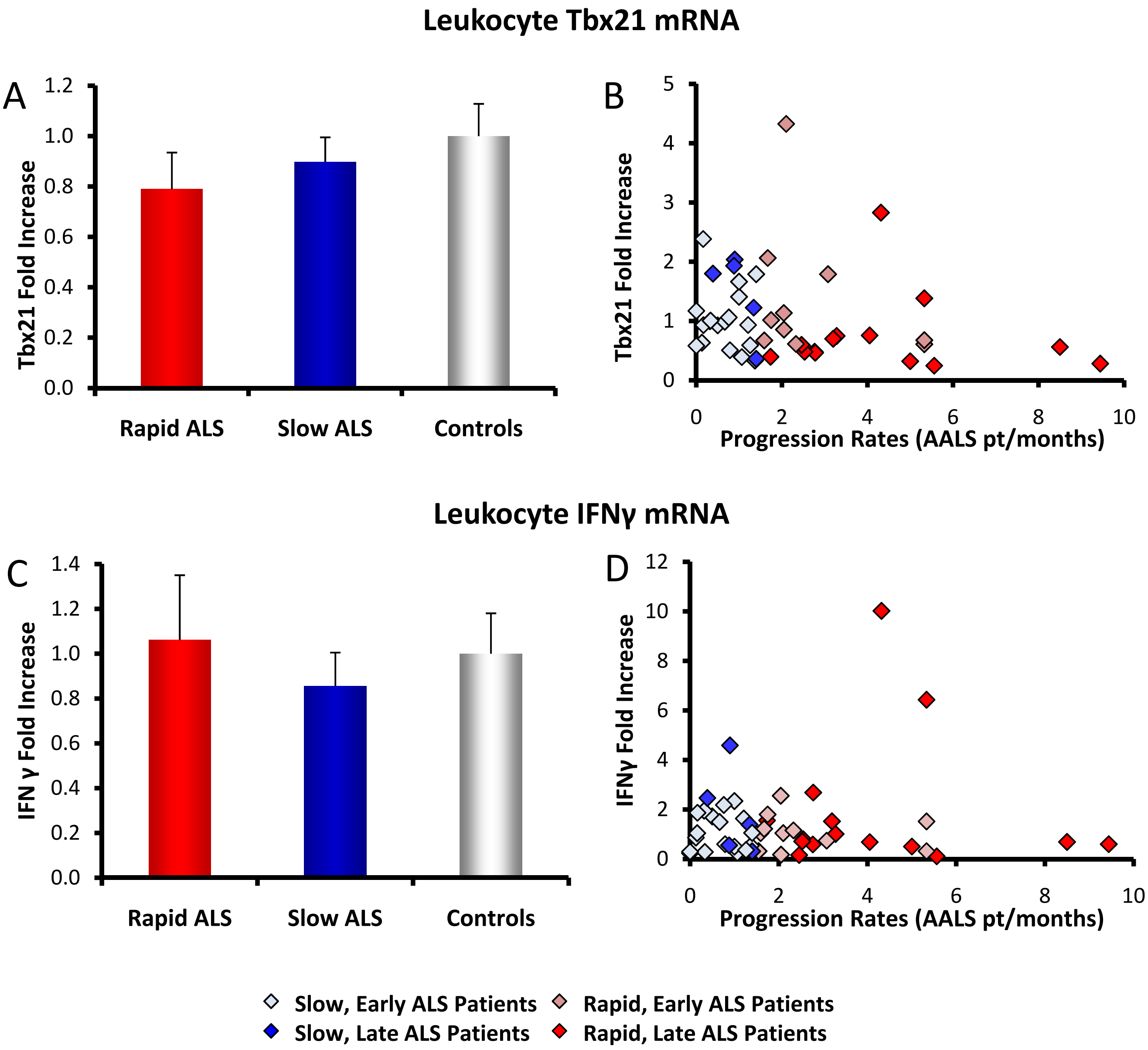


**Supporting Information Figure 3**
Leukocyte Tbx21 (Tbet) and IFNγ mRNA expression levels are not different in ALS patients regardless of the progression rate. qRT-PCR was utilized to evaluate expression levels of Tbox21 mRNA isolated from leukocytes of ALS patients and control volunteers. (A, B) No differences were found in Tbx21 mRNA expression in ALS patients regardless of progression rate (T-test), and Tbx21 did not correlate with disease progression rate (R=0.199: linear regression). (C, D) No differences were found in IFNγ mRNA expression in ALS patients regardless of progression rate (T-test), and IFNγ did not correlate with disease progression rate (R=0.101: linear regression).
